# Supplementary material for: Cohort study protocol of the Brazilian collaborative research network on COVID-19: strengthening WHO global data
Source: BMJ Open. 2022 Nov 2;12(11):e062169. doi: 10.1136/bmjopen-2022-062169 (PMC9638748; doi:10.1136/bmjopen-2022-062169)
Supplement: Author's manuscript [file bmjopen-2022-062169.draft_revisions.pdf]

BMJ Open is committed to open peer review. As part of this commitment we make the peer review history of every article we publish publicly available.

When an article is published we post the peer reviewers' comments and the authors' responses online. We also post the versions of the paper that were used during peer review. These are the versions that the peer review comments apply to.

The versions of the paper that follow are the versions that were submitted during the peer review process. They are not the versions of record or the final published versions. They should not be cited or distributed as the published version of this manuscript.

BMJ Open is an open access journal and the full, final, typeset and author-corrected version of record of the manuscript is available on our site with no access controls, subscription charges or pay-per-view fees (http://bmjopen.bmj.com).

If you have any questions on BMJ Open's open peer review process please email info.bmjopen@bmj.com

BMJ Open

Brazilian network for collaborative research on COVID-19: strengthening WHO global data

Journal:	*BMJ Open*
Manuscript ID	bmjopen-2022-062169
Article Type:	Protocol
Date Submitted by the Author:	09-Mar-2022
Complete List of Authors:	Anschau, Fernando; Grupo Hospitalar Conceição, Research Sector<br>Aredes, Natália; Universidade Federal de Goiás, Nursing School; Brazilian Company of Hospital Services<br>Reveiz, L; Pan American Health Organization, Evidence and Intelligence for Action in Health<br>Padilla, Monica; Pan American Health Organization Brazil<br>Gomes, Rosane ; Pan American Health Organization Brazil<br>Carvalho , Wellington ; Pan American Health Organization Brazil<br>Leles, Fernando ; Pan American Health Organization Brazil<br>Reese, Fernanda; Complexo Hospitalar do Trabalhador<br>Hubert, André ; Complexo Hospitalar do Trabalhador<br>Kemper, Elisandréa ; Hospital da Criança de Brasília<br>de Souza , Renilson; Hospital da Criança de Brasília<br>Salviano, Cristiane ; Hospital da Criança de Brasília<br>e Silva, Hevelin ; Hospital da Criança de Brasília<br>Coelho, Eduardo; Brazilian Company of Hospital Services<br>Gatto, Giuseppe ; Brazilian Company of Hospital Services<br>de Morais, Rafael; Brazilian Company of Hospital Services<br>Alegre, Leonardo; Brazilian Company of Hospital Services<br>Padilha dos Reis, Rodrigo ; Universidade Federal do Rio Grande do Sul, Graduate Program in Epidemiology ; Hospital de Clínicas de Porto Alegre<br>dos Santos Neto , Joaquim; Universidade Federal do Rio Grande do Sul, Graduate Program in Epidemiology; Hospital de Clínicas de Porto Alegre<br>Purper, César; Grupo Hospitalar Conceicao Gerencia de Ensino e Pesquisa<br>dos Santos, Veridiana ; Grupo Hospitalar Conceição<br>Garbini, Andressa; Grupo Hospitalar Conceição<br>Charão de Almeida, Rafaela; Grupo Hospitalar Conceição<br>Donida, Bruna; Grupo Hospitalar Conceicao Gerencia de Ensino e Pesquisa<br>Bitencourt, Rogério; Grupo Hospitalar Conceição Gerência de Ensino e Pesquisa<br>Kopittke, Luciane; Grupo Hospitalar Conceição Gerência de Ensino e Pesquisa<br>dos Santos, Fernanda; Grupo Hospitalar Conceição<br>Lutkmeier, Raquel; Grupo Hospitalar Conceição<br>Carazai, Daniela ; Grupo Hospitalar Conceição<br>Reis , Virgínia ; Instituto de Saúde e Gestão Hospitalar<br>Deulefeu, Flávio ; Instituto de Saúde e Gestão Hospitalar<br>Severino, Fernanda; Instituto de Saúde e Gestão Hospitalar<br>da Costa Neto, José Gustavo ; Instituto de Saúde e Gestão Hospitalar

Carvalho, Nirvania ; Health Department of the State of Piauí, Hospital Getúlio Vargas
de Andrade, André ; Health Department of the State of Piauí, Hospital Getúlio Vargas
Teixeira, Adriana; Brazilian Ministry of Health, Department of Hospital, Home, and Emergency Care (DAHU) of the Specialized Health Care Office (SAES)
Braga Neto, Olavo ; Brazilian Ministry of Health, Department of Hospital, Home, and Emergency Care (DAHU) of the Specialized Health Care Office (SAES)
Muller, Gabriel ; Universidade Federal do Rio Grande do Sul, Graduate Program in Epidemiology; Hospital de Clínicas de Porto Alegre
Kuchenbecker, Ricardo; Universidade Federal do Rio Grande do Sul, Graduate Program in Epidemiology ; Hospital de Clínicas de Porto Alegre

Keywords: COVID-19, Epidemiology < INFECTIOUS DISEASES, Health policy < HEALTH SERVICES ADMINISTRATION & MANAGEMENT

SCHOLARONE™ Manuscripts

Brazilian network for collaborative research on COVID-19: strengthening WHO global data

Authors:

Fernando Anschau[1], Natália Del' Angelo Aredes[2], Ludovic Reveiz[3]; Monica Padilla[3]; Rosane de Mendonça Gomes[4]; Wellington Mendes Carvalho[4]; Fernando Antonio Gomes Leles[4]; Fernanda Baeumle Reese[5]; André Hostílio Hubert[5]; Elisandréa Sguario Kemper[6]; Renilson Rehem de Souza[6]; Cristiane Feitosa Salviano[6]; Hevelin Silveira e Silva[6]; Eduardo Barbosa Coelho[7]; Giuseppe Cesare Gatto[7]; Rafael Freitas de Morais[7]; Leonardo Nunes Alegre[7], Rodrigo Citton Padilha dos Reis[8], Joaquim Francisco dos Santos Neto[8], Andresa Fontoura Garbini[1]; Cesar Perdomo Purper[1], Veridiana Baldon dos Santos[1]; Rafaela dos Santos Charão de Almeida[1]; Bruna Donida[1]; Rogério Farias Bitencourt[1]; Luciane Kopittke[1]; Fernanda Costa dos Santos[1]; Raquel Lutkmeier[1]; Daniela dos Reis Carazai[1]; Virgínia Angélica Silveira Reis[9]; Flávio Clemente Deulefeu[9]; Fernanda Gadelha Severino[9]; José Gustavo da Costa Neto[9]; Nirvania do Vale Carvalho[10], André Jamson Rocha de Andrade[10]; Adriana Melo Teixeira[11]; Olavo Braga Neto[11], Gabriel Cardozo Muller[8], Ricardo de Souza Kuchenbecker[8]

[1] Hospital Nossa Senhora da Conceição – Grupo Hospitalar Conceição

[2] Nursing School, Universidade Federal de Goiás, member of the EBSERH research team

[3] Pan American Health Organization (PAHO) - World Health Organization (WHO)/Washington, DC, US

[4] PAHO/WHO in Brazil

[5] Complexo Hospitalar do Trabalhador

[6] Hospital da Criança de Brasília

[7] Brazilian Company for Hospital Services (Empresa Brasileira de Serviços Hospitalares - EBSERH)

[8] Graduate Program in Epidemiology UFRGS, Hospital de Clínicas de Porto Alegre

[9] Instituto de Saúde e Gestão Hospitalar

[10] Hospital Getúlio Vargas- Teresina, Piauí - Health Department of the State of Piauí

[11] Department of Hospital, Home, and Emergency Care (DAHU) of the Specialized Health Care Office (SAES) of the Brazilian Ministry of Health

For peer review only

Brazilian network for collaborative research on COVID-19: strengthening WHO global data

Corresponding author:

Fernando Anschau

Av. Francisco Trein n.:326

Bairro Cristo Redentor – Porto Alegre, Rio Grande do Sul,

Brazil Zip Code: 91350-200

e-mail: afernando@ghc.com.br

telefone number: +55 (51) 3357 2805

Word Count: 4366

For peer review only

Brazilian network for collaborative research on COVID-19: strengthening WHO global data

Abstract

Introduction: The COVID-19 pandemic has imposed a triple challenge on low-income countries hospitals. First, the large number of patients requiring hospitalization due to infection's more severe symptoms. Second, the absence of systematic and broad testing policies with early identification of cases. Third, the weaknesses in integration of information systems, which led to the need to search for available information from the hospital information systems. In this context, it is also important to state that relevant aspects of COVID-19's natural history have not yet been fully clarified. This research protocol aims to present the strategies of a Brazilian network of hospitals to perform systematized data collection on COVID-19 through the World Health Organization (WHO) Platform. **Methods:** This is a multicenter project among Brazilian hospitals to provide data on COVID-19 through the WHO global platform, which integrates patient care information from different countries. From October 2020 to March 2021, a committee worked on defining a flowchart for this platform, specifying the variables of interest, data extraction standardization, and analysis. **Discussion:** The strategy presented by the WHO enables knowledge advancement during the pandemic, recognizing changes in care patterns, and the different conditions between locations. Based on the results organization, it is possible to analyze the data from different locations in Brazil, which reflect in the health system the inequalities found in this continental country. **Ethics and Dissemination:** This protocol was approved by Research Ethics Committee and will have its results explained in WHO reports and scientific articles.

Keywords: COVID-19, clinical data, morbidity, mortality

For peer review only

Brazilian network for collaborative research on COVID-19: strengthening WHO global data

Article Summary

This is a multicenter project among Brazilian hospitals to provide data on COVID-19 through the WHO global platform.

It is expected to deepen knowledge about the pandemic scenario and help hospital institutions to develop preventive measures, health service protocols and strengthen the training of teams in the existing complications.

For peer review only

Introduction

COVID-19 is a disease caused by SARS-CoV-2, started in Wuhan (Hubei province, China) in December 2019 (1), and characterized as a pandemic by WHO on March 11, 2020 (2). The disease caused by SARS-CoV-2 has a lower lethality when compared to those of two other coronaviruses that have already provoked epidemics, such as SARS-CoV and MERS-CoV. However, its high transmission rate impacts on a relevant demand to health systems, with an increase in the number of hospitalizations, need for oxygen by assisted ventilation, and other forms of clinical support (3-5).

Brazil ranks third worldwide in the total number of COVID-19 cases, with all regions affected (6,7), and demographic and social inequalities impact the results obtained in transmission control and health care. Low- and middle-income countries, such as Brazil, had great difficulty adopting pandemic mitigating measures such as non-pharmacological interventions (8). Combined with the high transmissibility of variants of interest, so-called *variants of concern* (VOC), the pandemic's increased extent can be explained (9,10).

Brazil is a country of continental dimensions, with 6,820 hospitals, of which 2,423 are public hospitals and 4,397 are private hospitals (11). The country has 536,474 hospital beds (1.96 beds per 1000 inhabitants and 0.8 beds per 1000 inhabitants in public hospitals) (11,12). There are disparities in the availability of hospital beds in the different regions, including intensive care, which is of utmost importance for the care of COVID-19 patients. For every 100,000 inhabitants, there are 9.73 ICU beds in the Unified Health System (Sistema Único de Saúde - SUS) in the North region, 10.46 in the Northeast region, 11.87 in the Midwest region, 13.24 in the Southeast region, and 15.88 in the South region, according to data from 2020 (13).

Between December 2019 and April 2020, ICU beds were expanded from

For peer review only

46,045 to 60,265 (13) nationwide with an overall increase of 30.88%, with the increase in ICU beds under SUS being almost four times smaller than the increase in ICU beds in the private network (13.46% increase under SUS and 48.33% in the private sector), signaling a need for public investment, especially in view of the gaps identified during the pandemic.

A study that evaluated the first 250,000 SARS-Cov-2 hospitalizations in Brazil from the national Severe Acute Respiratory Syndrome case database (SIVEP-Gripe) highlighted the in-hospital mortality rate - which reached 38%, the number of people with demands compatible with Intensive Care Unit (ICU) (39%) and regional discrepancies, revealing considerable challenges in managing the pandemic in Brazil (14). However, the results presented a high fraction of missing data on some variables, especially comorbidities and symptoms, and non-standardized reporting protocols (14). Additionally, these data do not include the so-called "second wave", caused predominantly in Brazil by new highly transmissible variants such as P1 and B1.1.7 (15-17).

Data from the two waves observed in Brazil using the same dataset (SIVEP-Gripe) were published in 2021. The cross-sectional study of hospitalized patients with RT-PCR positive for SARS-CoV-2 in Brazil comprised data entered into SIVEP-Gripe from February 25, 2020 to April 30, 2021, separated into two waves on November 5, 2020. As opposed to what was seen in the rest of the world, in Brazil in-hospital mortality increased from 34.81% in the first wave to 39.30% in the second wave. In the second wave, there were more ICU admissions, use of non-invasive and invasive ventilation, and increased mortality in the younger age groups. The Brazilian South and Southeast regions had the highest hospitalization rates per 100,000 inhabitants. Nevertheless, the in-hospital mortality rate was higher in the Northern and Northeastern states. Racial differences were observed in clinical outcomes, with white people being the most prevalent hospitalized population,

For peer review only

though black/brown-skinned people had higher mortality rates. The younger age groups presented more considerable differences in mortality compared to the groups with and without comorbidities at both waves. The results reflect the inequalities observed in Brazil and the overload of health systems upon the second wave's arrival (18).

It is critical to maintain surveillance on the pandemic's evolution over time and across the country to measure its impact on the health care system. In Brazil, the Unified Health System (SUS) consists of a public network of services at all levels of care, on which about 75% of the population depends exclusively. Today, 66.34% of hospital beds belong to SUS (19), which characterizes the Brazilian public system as responsible for most of the health care during the COVID-19 pandemic in the country. Even so, both public and private hospitals do not use a unified electronic record system, which increases the heterogeneity of reports and represents an obstacle for data collection in research.

As a strategy to overcome the challenges of data collection for health research and management purposes, the Pan American Health Organization (PAHO)-World Health Organization (WHO) proposed a collaboration in 2020 between hospitals in all regions of Brazil to join databases and collect clinical data from COVID-19 patients. Through this initiative it is possible to aggregate data from different locations around the country, and the world, to characterize the disease's natural history, identify risk factors for severe cases, and describe treatment interventions and outcomes. Thus, it directly contributes to national and global public health with scientific evidence on the COVID-19 clinical management and the strengthening of research networks and technology-supported data collection.

As far as we know, this is the first initiative in Brazil to create a "Big Data" database in healthcare from hospitals and, afterwards, a *data hub* with clinical conditions and usage patterns from the healthcare system.

For peer review only - http://bmjopen.bmj.com/site/about/guidelines.xhtml

This study aims to standardize and provide quality clinical data on the COVID-

For peer review only

19 pandemic in Brazil. Furthermore, it aims to describe the resources and methods used in this collaboration to identify and validate the cases in each database containing cases confirmed as infected with SARS-CoV-2 by laboratory examination (case group) and suspects (control group).

Methods

This study is being conducted in 43 hospitals located in all regions of Brazil. From October 2020 to March 2021, a committee of experts worked on defining a blueprint for this platform, specifying variables of interest, data extraction standardization, and data analysis.

Aiming to standardize data collection, the project uses *case report forms* (CRF) to extract clinical information from adults and children hospitalized with COVID-19 (access from the platform URL: https://www.who.int/teams/health-care-prontidão-clínica-unidade/COVID- 19/data platform). The data from all the centers surveyed can be aggregated and analyzed - either together or by center or region of the country - to provide a better understanding of the disease and present the public health response to the pandemic. Collecting anonymized clinical data globally will provide WHO:

1. Identification of the main clinical characteristics and prognostic factors of hospitalization cases for suspected or confirmed COVID-19, expanding the knowledge on the severity, spectrum, and impact of the disease on the hospitalized population globally and in different countries.
2. Identification of clinical interventions, facilitating global and country operational planning during the COVID-19 pandemic.

The research questions and hypotheses of the Brazilian protocol of the Global Clinical Platform on COVID-19 are presented in Table 1 below.

For peer review only - http://bmjopen.bmj.com/site/about/guidelines.xhtml

9

Table 1 - Project research questions and hypotheses	
**Questions**	**Hypotheses**
Are the descriptions presented in the international literature regarding infections by the new coronavirus 2019 applicable to the Brazilian reality?	The descriptions presented in the existing academic literature on the clinical, laboratory, radiological, and therapeutic profile of patients diagnosed with the new coronavirus 2019 and admitted to Brazilian hospitals are different, a factor that makes it essential to consider Brazilian peculiarities during this disease's screening, diagnosis, and treatment.
What are the main symptoms presented by Brazilian patients with SARS- CoV-2 infection, especially those admitted to different hospital units in Brazil?	The most common clinical manifestations in Brazilian patients tested for coronavirus are related to fever, myalgia, respiratory symptoms, and headache.
Is in-hospital mortality from COVID-19 similar in Brazil and the rest of the world?	The in-hospital mortality profile of patients admitted to Brazilian hospitals with COVID-19 is not similar to that presented in the literature.

For peer review only

The primary and secondary objectives of the Brazilian protocol of the Global Clinical Platform on COVID-19 are:

- Primary objective: To determine the clinical, laboratory, and radiological profile, therapeutic practice, and mortality of patients confirmed with new coronavirus 2019 infection admitted to Brazilian hospitals.
- Secondary objectives: 1) To identify outcomes such as intensive care unit (ICU) admission, length of hospital stay, duration on mechanical ventilation and respiratory therapy; 2) To assess the incidence of adult respiratory dysfunction syndrome and need for mechanical ventilation and intensive care, and days of intensive care in patients admitted to hospitals; 3) To identify the incidence of signs and symptoms of multisystemic inflammatory syndrome and associated chronic diseases.

Study design, population, and sample

This is a multicenter cohort study with convenience sampling from the population of patients hospitalized with COVID-19, either by clinical suspicion or laboratory confirmation. As inclusion criteria for cases, patients with confirmed COVID-19 (RT-PCR tests or rapid tests), admitted to hospitals with a minimum length of stay of 24 hours, are considered.

Main outcomes and parameters

The CRFs were developed by WHO for the collection and submission of harmonized data to the COVID-19 Global Clinical Platform, and the procedures for further data collection were performed by the committee's Technical Team.

Key descriptive parameters include socio-demographic data (age, gender), presence of preexisting conditions, use of chronic medications, clinical characteristics on admission and during hospitalization, laboratory findings on admission and during hospitalization, clinical interventions on admission and during hospitalization (use of oxygen, use of mechanical ventilation, use of therapeutics),

For peer review only

and patient outcomes (death, discharge, referral).

The patient outcomes described above will be used in secondary analysis to investigate associations between baseline characteristics and disease severity and outcomes.

Statistical considerations

General considerations

Statistical analyses (numerical summaries, point estimates, confidence intervals, hypothesis tests, fitting regression models, tables, graphs, and maps) will be performed in R[3] version 4.0.5 or higher. Specification of sets of adjustment variables in certain association analyses will be conducted in DAGitty [4] software.

Missing Data

For each analysis, the denominator will represent the number of available observations.

The date of hospital admission will be considered fundamental and critical to know the hospitalized population to be analyzed. Records with missing admission dates will be excluded from the analysis unless there is confirmation from participating hospitals that the data refer to a cohort of hospitalized patients.

Data imputation will not be performed, but can be considered for each particular analysis.

Descriptive statistics

According to the type of variable, the descriptive statistics will be:

- Quantitative variables: number of observations (N), mean, standard deviation (SD), median, and interquartile range (IQR), depending on the distribution of the variable.
- Qualitative variables: number of observations (N), absolute frequency (n), and relative frequency (%).

For peer review only

- Percentages will be calculated on the number of participants with observed data (n/N).

Objectives of the analysis (See Supplementary 1)

1. Description of socio-demographic and clinical characteristics

Describe the socio-demographic and clinical characteristics of hospital admissions, interventions, and clinical outcomes among:

- people infected with the SARS-CoV-2 virus cared for by hospitals within the Global Platform;
- specific population groups, such as children, pregnant women, populations with co-infections, comorbidities, among others.

2. Characterization of access profiles and hospital resource utilization patterns

Describe access profiles and hospital resource utilization patterns such as emergency care, inpatient admissions in wards and intensive care units, including non-invasive and invasive ventilation devices, and non-traditional care modalities such as intermediate units, ventilatory support, among others.

3. Association between clinical/socio-demographic characteristics and outcomes

Investigate factors associated with the disease severity, ICU admission, in-hospital mortality among:

- people infected with the SARS-CoV-2 virus cared for by hospitals within the Global Platform;
- specific population groups, as described above.

4. Sources of inequities

Investigate potential sources of inequities in timely access to diagnostic and therapeutic services and resources and the association with clinical outcomes.

For peer review only

Data governance

Defining data governance is always necessary in a research protocol, considering that data is protected by legislation, including the rights of research participants. This model dictates that the hospitals involved understand the research aspects, the information security points, and complete the process of sending data to the central repository in a clear, unambiguous manner. Furthermore, regarding information security, the central repository's teams are required to follow procedures ensuring ethical and secure actions, in compliance with the legislation in force, when processing information.

Privacy and Consent Policies are important points for discussion on health data sharing. Therefore, for the preparation of this proposal, the concepts and technical principles of the General Data Protection Act (LGPD) were respected, considering health data as sensitive, as described in these documents (20). The LGPD regulates the processing of personal data and aims to protect citizens' right to freedom, privacy, and free development.

Considering all these aspects, three levels of governance/access were defined: strategic, tactical, and operational:

1. The Advisory Committee of the PAHO/WHO COVID-19 Platform Project represents the first, whose main objective is to technically support PAHO/WHO in the terms of the COVID-19 Global Clinical Data Platform, and the participating institutions in the activities of preparation for analysis and publication of clinical and epidemiological data;
2. The second consists of those responsible for translating the strategy and integrating all parties, setting the objectives, and managing the project, including defining the work processes, schedule, team engagement and communication, quality, and results follow-up;
3. The third performs activities from the tactical level always in compliance with data

For peer review only - http://bmjopen.bmj.com/site/about/guidelines.xhtml

security standards and rules. It is at this level that statements like "data must be stored securely" translate into technological tools ensuring the semantics read into the sentence.

Risks

The risks are related to the loss of confidentiality. However, they have been and will be constantly mitigated/controlled by the anonymization of the participants' identification data. Researchers take responsibility for maintaining their commitment to preserve the data's security and confidentiality, and hospital institutions are asked to take all necessary steps to protect their access credentials and passwords to the COVID-19 Data Platform.

The collected and anonymized data will be stored by the aforementioned WHO Platform, a secure, password-protected, restricted-access electronic platform. WHO commits to: i) protect the confidentiality of anonymous data on COVID-19 and prevent its unauthorized disclosure; ii) implement and maintain technical and organizational security measures to protect the security of anonymous data on the Platform; and iii) follow the International Health Regulations (21).

Benefits

It is expected that this protocol will develop specific and reliable knowledge on the COVID-19 pandemic - clinical and epidemiological analysis; discussing the Brazilian and other countries' reality and plans to fight the pandemic. It is expected that it will deepen the knowledge on the pandemic scenario and help hospital institutions to develop preventive measures, health service protocols, and strengthen staff training on existing complications. The information generated by the research can serve as a basis for developing maps of the SARS-CoV-2 infection evolution.

Clinical data extraction

As the WHO representative in the country, the PAHO/WHO office in Brazil, through the Technical Unit of Health Systems and Services, coordinated the

For peer review only - http://bmjopen.bmj.com/site/about/guidelines.xhtml

project by dialoguing with the partner institutions to make this project effective. In this context, Grupo Hospitalar Conceição (GHC) became the national coordinating Brazilian institution for submission procedures to the Research Ethics Committee (CEP) and the National Research Ethics Commission (CONEP).

Data collection started retrospectively in 43 Brazilian hospitals - as shown in Figure 1 - starting from the first COVID-19 case diagnosed in Brazil (March 2020) and ending in March 2021. We chose to carry out this first stage of data collection directly in the hospital units, consulting the databases of the clinical and care information systems available in each institution or the physical records of the hospitalizations of suspected or confirmed patients with COVID-19 in these units.

The selected hospitals are responsible for providing all the conditions for the project's execution, such as access to data collection and feeding the platform to the PAHO/WHO consultant assigned to each institution. In order to do so, each hospital signed a commitment term, which contains all the guarantees offered by the WHO to maintain the data confidentiality and anonymization. The collection and access process, agreed with each hospital unit, respects the specificities and internal regulations, and also follows the ethical and data security norms.

Considering that the information contained in the hospital records of each institution can be divided between structured and open text, and in view of the heterogeneities of Brazilian hospitals in terms of management, there is a need to organize data collection in several ways: one through manual consultation of electronic citizen records (ECR) - or physical records for hospitals that do not have ECRs - and filling out the WHO-specific forms; another through the development of an automated programming interface (API) for filling out these forms.

The standardized API was developed by information technology (IT) professionals to share databases from different hospitals. These databases, with provision for secure cloud-based storage, contain de-identified electronic health records (EHRs) corresponding to admission notes for the first 48 hours of care and

For peer review only

the 24 hours prior to discharge/outcome for each research participant.

The structured ECR data contains demographic information, prescriptions, lab results, vital signs, and general patient characterization such as height and weight, admission and outcome dates. Since the lack of data for different variables can be significant, there is the possibility for institutions to search and fill in part of the variables from unstructured data by open text extraction.

For this procedure, the Smart Health Connect (SHC) software is used, which operates based on an algorithm using Deep Neural Networks, extracting information from the clinical evolutions and incorporating data to the CRF that were previously missing, reinforcing the completeness of the database exported to the WHO. The main variables of interest incorporated through this software were comorbidities, symptoms, clinical events, and especially those related to COVID-19, such as oxygen support and ventilation type, and outcomes. The software is capable of recognizing and categorizing data regarding history (past), current events (present), and expected actions or outcomes (future); and identifying variables related to drug treatment, clinical condition, including vital signs, symptoms, clinical devices, affections/pathologies, and other relevant data that could not be captured in the structured ECR data fields.

SHC uses the newest natural language processing techniques available to the scientific community and a software architecture model that enables mass data extraction.

Regarding the technological standard used by the software, there are two approaches supported by artificial intelligence. The first is Named Entity Recognition - NER, which has the function of locating previously defined entities in open text. For this project, the categories are disease names, signs and symptoms, procedures, medications, ventilatory support, and test results. SHC identifies and marks in the text each of the identified categories in a process known as inference.

For peer review only

Once the relevant terms are identified, the other step is the analysis of the findings in a given text, called Assertion Detection. Through this approach it is possible to differentiate the status of what has or has not actually occurred with that patient. This is necessary because the medical record can indicate something related to the past, or to a third party, such as family history, or even state that the patient does not have a certain condition, or does not use a certain medication.

Expected results

At the end of the project, in addition to the reports produced by WHO with the data provided globally and the ensured access to the databases, PAHO/WHO in Brazil will organize publications in partnership with the institutions participating in the project in the country, highlighting the results and Brazil's contribution in fighting the COVID-19 pandemic.

Based on this protocol design, it will be possible to continue data collection among the participating hospitals and continuously add valuable information for public health in the context of facing the pandemic in Brazil. Once collaboration between institutions has taken place and the form of data collection, the variables of interest, and the technologies to support the search for information of interest have been standardized, there is significant potential to advance the analysis of evidence and response measures in the national health system.

Until now, the identification of this strategy's partner hospitals and their characterization, with number and type of beds, brings an analysis perspective of the health care network scenario in the fight against the pandemic (Table 2).

Funding

The WHO Global Clinical Platform for COVID-19 is supported by WHO through an R&D grant from Germany.

Ethics

This protocol was approved by the Research Ethics Committee (CEP) of the

For peer review only
For peer review only - http://bmjopen.bmj.com/site/about/guidelines.xhtml

Research Coordinating Center of Brazil (CEP of the Hospital Nossa Senhora da Conceição), on January 29, 2021, under opinion number: 4.515.519 and by the National Research Ethics Commission (CONEP), on February 5, 2021, under opinion number: 4.526.456. Afterwards, it had the approval of the local CEPs of each institution involved. In addition, this study followed the resolutions 466/12 and 510/16 and the resolutions CNS No. 466/2012 and No. 510/2016 of the National Health Council, which provide on the ethical aspects involving the use of data directly obtained from participants or identifiable information, or that may entail risks greater than those existing in everyday life.

Strengths and Limitations

Through this initiative it is possible to aggregate data from different locations around the country to characterize the disease's natural history, identify risk factors for severe cases, and describe treatment interventions and outcomes. Thus, it directly contributes to national and global public health with scientific evidence on the COVID-19 clinical management and the strengthening of research networks and technology-supported data collection.

As far as we know, this is the first initiative in Brazil to create a "Big Data" database in healthcare from hospitals and, afterwards, a *data hub* with clinical conditions and usage patterns from the healthcare system.

Because this is a convenience, non-probabilistic sample of patients admitted to health care facilities it may not represent the population of COVID-19 patients hospitalized in the country. However, the information generated by this research can serve as a basis for the development of maps of the evolution of SARS-CoV-2 infection and public policies to face pandemics.

Patient and Public Involvement

This project is already involving both Brazilian communities and public agencies that are facing the fight against the Covid-19 pandemic, as well as the Brazilian Ministry

For peer review only

of Health and PAHO/WHO. We intend to expand partnerships and involve more stakeholders from Brazil.

Discussion

One of the main concerns regarding the COVID-19 pandemic is its burden on the health care system organization (22). The need for a general and effective organization of the health services to treat the population safely and fight the disease's complications has required a great deal of effort. This includes reallocating health care workers, using appropriate medicines, producing, distributing, and delivering vaccines to the population, and increasing health care capacity (23).

The outbreak of the highly transmissible delta variant (B.1.617.2) raises new questions among policy makers and health system managers regarding the impact on hospitalization rates, a point reinforced by a recent research publication that indicates a possible worst-case scenario compared to the alpha variant (B .1.1.7) (24). Additionally, according to Twohig KA et al. (2021), this recent research adds to previous evidence and reinforces that the delta variant, in unvaccinated populations, may increase the burden to the overall health care system when compared to the SARS-CoV-2 alpha variant (24).

The Brazilian ICU occupancy condition became critical in most of the territory in March 2021, when 20 states reached critical rates (equal to or greater than 80% occupancy) and became intermediate in most states by June 2021 (between 60 and 80%), in concurrence with high SARS-CoV-2 transmission rates (25,26).

It is necessary to maintain biosafety education for the general population (social distance and the use of masks), encourage them to seek the vaccine when called upon by age/group, and complete the recommended number of doses. At the organizational level of hospitals, it is critical to measure resources and indicators, planning strategies according to the literature and lessons learned during the pandemic to meet the next demand. For the public policy level, it is crucial to

For peer review only

For peer review only - http://bmjopen.bmj.com/site/about/guidelines.xhtml

intensify vaccination throughout the country and support the health system by consolidating its structure and strategies for serving the population.

The initial ignorance regarding the virus and the associated disease led hospitals and health services to seek different internal management strategies to meet the demand coming with COVID-19 (27). Berwick DM used the term "choices for the new normal" to point to six properties of care for lasting change: time, standards, working conditions, proximity, readiness, and equity. For the author, the changes in care required by the pandemic may allow health systems to become more patient-centered, efficient, and consequently provide better care to the population (28).

Since the spectrum of SARS-CoV-2 infection can range from asymptomatic infection to potentially fatal complications of COVID-19, we chose to list patients with suspected and confirmed infection who were admitted to hospital units (29). Thus, we believe that the strategy of the "COVID-19 Global Clinical Data Platform for clinical characterization and management of hospitalized patients with suspected or confirmed COVID-19" contributes to the understanding of the Brazilian context of pandemic crisis in the healthcare system by providing data analysis on clinical aspects and epidemiology.

It also reinforces a portrait of the reality of the institutions based on the current demand, emerging the discussion on aspects of the main organizational frameworks for fighting the pandemic and how to keep the institutions functioning and meeting the health needs of the population amidst the challenges.

For peer review only

Conclusions

This project describes the collaboration of several Brazilian hospitals to build a joint database, with special attention to protocols aimed at reducing heterogeneity between different platforms and ensuring the quality of research data.

As part of the process, the project management carried out an organized multicenter research framework involving PAHO/WHO, as the center of the strategy, and Brazilian public hospitals, resulting in significant data collection. In addition, the data extraction used innovative techniques in the technology area, promoting quality and mitigating the lack of relevant information, moving towards data collection in unstructured fields, such as clinical evolution which are the texts with the most detailed health data in the medical records.

This proposal accomplishes an important step towards fighting COVID-19 in a developing country of continental dimensions such as Brazil, by building a foundation for new collaborations between referral hospitals and demonstrating the feasibility of joint initiatives for the emergence of "big data" analysis strategies.

Acknowledgements

This project received the institutional support from the hospital institutions mentioned here through the access to data and research participants, which were critical for this research, the reason for our gratitude.

The authors would like to thank the research participants which made the development of the research project possible.

Conflict of interests

The authors declare that they have no conflict of interest with the subject of the referred protocol.

For peer review only

References

1. Wu F, Zhao S, Yu B, Chen YM, Wang W, Song ZG, et al. A new coronavirus associated with human respiratory disease in China. Nature. 2020;579(7798):265–9.

2. World Health Organization. Coronavirus Disease 2019. Situat Rep [Internet]. 2020;54:1–9. Available from: https://www.who.int/docs/default-source/coronaviruse/situation-reports/20200314-sitrep-54-covid-19.pdf

3. Roussel Y, Giraud-gatineau A, Jimeno M, Rolain J. SARS-CoV-2: fear versus data. nternational J Antimicrob Agents. 2020;55(January):1–3.

4. Shen Y, Cui Y, Li N, Tian C, Chen M, Zhang YW, et al. Emergency Responses to Covid-19 Outbreak: Experiences and Lessons from a General Hospital in Nanjing, China. Cardiovasc Intervent Radiol [Internet]. 2020;43(6):810–9. Available from: https://doi.org/10.1007/s00270-020-02474-w

5. Li T. Diagnosis and clinical management of severe acute respiratory syndrome Coronavirus 2 (SARS-CoV-2) infection: an operational recommendation of Peking Union Medical College Hospital (V2.0): Working Group of 2019 Novel Coronavirus, Peking Union Medical Colle. Emerg Microbes Infect. 2020;9(1):582–5.

6. Cimerman S, Chebabo A, Cunha CA da, Rodríguez-Morales AJ. Deep impact of COVID-19 in the healthcare of Latin America: the case of Brazil. Brazilian J Infect Dis. 2020;24(2):93–5.

7. Barberia LG, Gómez EJ. Political and institutional perils of Brazil's COVID-19 crisis. Lancet. 2020;396(10248):367–8.

8. Gonçalves MR, Padilha dos Reis RC, Tólio RP, Pellanda LC, Schmidt MI, Katz N, et al. Social distancing, mask use, and transmission of severe acute respiratory syndrome Coronavirus 2, Brazil, April–June 2020. Emerg Infect Dis. 2021;27(8):2135–43.

For peer review only

9. Flaxman S, Mishra S, Gandy A, Unwin HJT, Mellan TA, Coupland H, et al. Estimating the effects of non-pharmaceutical interventions on COVID-19 in Europe. Nature. 2020;584(7820):257–61.

10. Walker PGT, Whittaker C, Watson OJ, Baguelin M, Winskill P, Hamlet A, et al. The impact of COVID-19 and strategies for mitigation and suppression in low- And middle-income countries. Science (80-). 2020;369(6502):413–22.

11. Federação Brasileira de Hospitais, Confederação Nacional de Saúde. Cenário dos hospitais no Brasil 2018 [Internet]. 2018. 108 p. Available from: http://fbh.com.br/wp-content/uploads/2020/11/1-Relatorio-FBH-CNS_web.pdf

12. Ministério da Saúde B. Cadastro Nacional de Estabelecimentos de Saúde [Internet]. 2021. Available from: http://cnes.datasus.gov.br/Cabecalho_Reduzido_Competencia.asp?VCod_Unidade=3205304044916

13. Cotrim Junior DF, Cabral LM da S, Machado FR de S. Fila única de leitos: urgência, dificuldades e enfrentamentos. P2P E Inovação. 2020;7:212–29.

14. Ranzani OT, Bastos L, Gelli J, Marchesi J, Baião F, Hamacher S, et al. Characterisation of the first 250 000 hospital admissions for COVID-19 in Brazil: a retrospective analysis of nationwide data. Lancet Respir Med 2021; 2020;9(January):407–18.

15. Claro IM, Cristina F, Ramundo MS, Candido DS, Silva CAM, Jesus JG De, et al. Local Transmission of SARS-CoV-2 Lineage B.1.1.7, Brazil, December 2020. Emerg Infect Dis [Internet]. 2021;27(3):2020–2. Available from: www.cdc.gov/eid

16. da Silva MS, Demoliner M, Hansen AW, Gularte JS, Silveira F, Heldt FH, et al. Early detection of sars-cov-2 p.1 variant in southern brazil and reinfection of the same patient by p.2. Rev Inst Med Trop Sao Paulo. 2021;63:1–8.

17. Franceschi VB, Caldana GD, de Menezes Mayer A, Cybis GB, Neves CAM,

For peer review only

Ferrareze PAG, et al. Genomic epidemiology of SARS-CoV-2 in Esteio, Rio Grande do Sul, Brazil. BMC Genomics. 2021;22(1):1–17.

18. Zeiser FA, Donida B, da Costa CA, Ramos G de O, Scherer JN, Barcellos NT, et al. First and second COVID-19 waves in Brazil: A cross-sectional study of patients' characteristics related to hospitalization and in-hospital mortality. Lancet Reg Heal - Am. 2022;6(November 2021):100107.

19. Brasil M da S do. DATASUS - Ministério da Saúde [Internet]. Datasus. 2021 [cited 2021 Aug 30]. Available from: www.datasus.gov.br

20. Brasil P da R. Lei Geral de Proteção de Dados Pessoais (LGPD). Diário oficial da união [Internet]. 2018; Available from: file:///C:/Users/usuario/Desktop/L13709compilado.html

21. Merianos A, Peiris M. International Health Regulations (2005). Lancet. 2005;366(January):1249–51.

22. Bartsch SM, Ferguson MC, McKinnell JA, O'shea KJ, Wedlock PT, Siegmund SS, et al. The potential health care costs and resource use associated with COVID-19 in the United States. JAMA J Am Med Assoc. 2020;39(6):927–35.

23. Glied S, Levy H. The Potential Effects of Coronavirus on National Health Expenditures. JAMA - J Am Med Assoc. 2020;323(20):2001–2.

24. Twohig KA, Nyberg T, Zaidi A, Thelwall S, Sinnathamby MA, Aliabadi S, et al. Hospital admission and emergency care attendance risk for SARS-CoV-2 delta (B.1.617.2) compared with alpha (B.1.1.7) variants of concern: a cohort study. Lancet Infect Dis. 2021;3099(21):1–9.

25. Fiocruz. Observatório Covid-19 [Internet]. https://portal.fiocruz.br/. 2021 [cited 2021 Aug 30]. p. 5. Available from: https://portal.fiocruz.br/sites/portal.fiocruz.br/files/documentos/boletim_covid_2021_extraordinario_30dejunho.pdf

For peer review only

26. Fiocruz. Leitos Uti Covid-19 Para Adultos [Internet]. https://portal.fiocruz.br/. 2021. p. 1. Available from: https://portal.fiocruz.br/sites/portal.fiocruz.br/files/documentos/serie_historica_leitos_uti_covid-19_adultos.pdf

27. Kim MK, Rabinowitz LG, Satish Nagula M, Dunn A, Chalil J, Xu T, et al. A Primer for Clinican Deployment to the Medicine Floors from an Epicenter of Covid-19. NEJM Catal. 2020;

28. Berwick DM. Choices for the “new Normal.” JAMA - J Am Med Assoc. 2020;323(21):2125–6.

29. Ortiz-prado E, Simbaña-rivera K, Barreno LG-, Rubio-neira M, Cevallos-robalino D, Sanches-sanmiguel H, et al. Clinical, molecular, and epidemiological characterization of the SARS-CoV-2 virus and the Coronavirus Disease 2019 (COVID-19), a comprehensive literature review. Diagn Microbiol Infect Dis. 2020;98(115094):1–31.

For peer review only

Table 2 - WHO Global Clinical Platform for COVID-19 characterization in Brazil: collaborating centers

STATE	Hospital Institution	Hospital beds							
		Surgical	Clinical	Complementary	Obstetrical	Pediatric	Medical Specialties	Hospital Day	Total
AM	HU Getúlio Vargas (HUGV-UFAM)	58	59	32	0	0	0	0	149
PA	Hospital Universitário João de Barros Barreto (HUJBB-UFPA)	40	178	38	0	28	12	10	306
CE	HU Walter Cantídio (HUWC-UFC)	68	72	21	0	20	22	4	207
CE	Maternidade Escola Assis Chateaubriand (MEAC-UFC)	21	58	65	104	6	0	0	254
CE	Hospital Regional Norte (HRN-ISGH)	58	183	177	33	70	16	0	537
CE	Hospital Geral Waldemar Alcântara (HGWA-ISGH)	18	188	58	0	33	33	0	330
CE	Hospital Regional do Cariri (HRC-ISGH)	88	112	113	0	0	29	0	342
CE	Hospital Regional do Sertão Central (HRSC-ISGH)	58	104	106	30	1	14	0	313
CE	Hospital Estadual Leonardo Da Vinci (Helv-ISGH)	1	112	179	0	0	0	0	292
CE	Hospital Regional Vale do Jaguaribe (HRVJ-ISGH)	0	30	10	0	0	0	0	40
DF	HU da Universidade de Brasília (HUB-UnB)	49	76	41	30	20	3	12	231
ES	HU Cassiano Antônio Moraes (HUCAM-UFES)	82	90	55	20	24	0	2	273
MA	HU da Universidade Federal do Maranhão	134	75	81	71	92	0	0	453
MT	HU Julio Muller (HUJM-UFMT)	12	20	31	24	11	0	0	98
MS	HU Maria Aparecida Pedrossian (Humap-UFMS)	60	45	53	32	18	0	12	220
MS	HU da Univ. Federal da Grande Dourados (HU-UFGD)	29	41	59	25	35	1	0	190
MG	HC da Univ. Federal do Triangulo Mineiro (HC-UFTM)	93	86	57	22	42	2	8	310
MG	HU da Univ. Federal de Juiz de Fora (HU-UFJF)	19	61	29	0	17	0	17	143
MG	HC da Univ. Federal de Minas Gerais (HC-UFMG)	119	114	107	35	63	1	30	469
PB	HU Lauro Wanderley (HULW-UFPB)	31	80	43	36	22	0	33	245
PE	HU da Univ. Fed. Vale do S. Francisco (HU-UNIVASF)	86	35	21	0	0	8	0	150
PE	HC da Univ. Federal de Pernambuco (HC-UFPE)	118	119	27	30	38	13	76	421
RJ	HU Antônio Pedro (HUAP-UFF)	83	54	59	21	17	0	10	244
RN	HU Ana Bezerra (HUAB-UFRN)	8	6	10	33	10	0	0	67
RS	HE da Univers. Feder. de Pelotas (HE-UFPel)	8	109	31	21	20	0	10	199
RS	HU da Univ. Federal de Santa Maria (HUSM-UFSM)	75	163	60	35	41	1	10	385

For peer review only

**SP**	**HU da Univ. Federal de São Carlos (HU-UFSCAR)**	0	56	12	0	17	0	0	85
**PB**	**HU Alcides Carneiro (HUAC-UFCG)**	29	84	31	0	33	0	0	177
**PB**	**HU Julio Bandeira (HUJB-UFCG)**	12	2	1	0	15	0	2	32
**RJ**	**HU Gaffree e Guinle (HUGG-UNIRIO)**	95	89	38	19	6	2	3	252
**SE**	**HU da Universidade Federal de Sergipe (HU-UFS)**	49	48	25	0	14	10	10	156
**SE**	**Hospital Universitário de Lagarto (HUL-UFS)**	25	46	33	0	16	0	10	130
**TO**	**Hospital de Doenças Tropicais (HDT-UFT)**	0	37	2	0	9	0	6	54
**BA**	**Hospital Especializado Octávio Mangabeira**	23	105	28	0	17	42	1	216
**PR**	**Complexo Hospitalar do Trabalhador**	126	37	132	35	30	24	0	384
**PR**	**Hospital do Trabalhador**	4	22	0	2	4	0	0	32
**PR**	**Hospital Osvaldo Cruz**	0	29	0	0	1	4	8	42
**DF**	**Hospital da Criança de Brasília José Alencar**	0	0	58	0	150	2	8	218
**PI**	**Hospital Getúlio Vargas**	170	87	94	0	0	2	11	364
**RS**	**Hospital Nossa Senhora da Conceição - Grupo Hospitalar Conceição**	207	357	332	62	105	30	13	1106
**RS**	**Hospital de Clínicas de Porto Alegre - HCPA/UFRGS**	228	224	259	50	95	30	17	903
**SP**	**Instituto de Infectologia Emilio Ribas, São Paulo**	4	82	232	0	16	10	31	375
**SP**	**Hospital Nestor Goulart Reis Americo Brasiliense**	0	20	0	0	0	73	0	93
**Total**		2388	3595	2840	770	1156	384	354	11487

STATE; AM: Amazonas; PA: Pará; CE: Ceará; DF: Distrito Federal; PB: Paraíba; PI: Piauí; RS: Rio Grande do Sul; RN: Rio Grande do Norte; SP: São Paulo; RJ: Rio de Janeiro; BA: Bahia; TO: Tocantins; SE: Sergipe; PA: Pará; PE: Pernambuco; ES: Espírito Santo; MA: Maranhão; MT: Mato Grosso; MS: Mato Grosso do Sul; MG: Minas Gerais.

HU: University Hospital; HE: Teaching Hospital

Source: The Brazilian National Register of Health Service Providers (CNES) (13).

For peer review only

Data Statment Section

Data from all surveyed centers can be aggregated and analyzed – together or by country center or region – to provide a better understanding of the disease and present the public health response to the pandemic via the platform URL: https://www.who.int/teams/health-care-clinic-readiness-unit/COVID-19/dataplatform

For peer review only

Author Statement

- Fernando Anschau[1],

  The author contributed to the organization of the group of researchers, data collection, analysis and writing of the protocol.
- Natália Del' Angelo Aredes[2]

  The author made substantial contributions to the design of work, data collection based on the protocol (in progress), literature review and writing. Revised the manuscript and have approved the submitted version.
- Ludovic Reveiz[3];

  The aforementioned author worked in the organization of the group of researchers and logistical support.
- Monica Padilla[3];

  The aforementioned author worked in the organization of the group of researchers and logistical support.
- Rosane de Mendonça Gomes[4];

  Logistical support and data analysis.
- Wellington Mendes Carvalho[4];

  Logistical support and data analysis.
- Fernando Antonio Gomes Leles[4];

  Logistical support and data analysis.
- Fernanda Baeumle Reese[5];

  Database organization and data collection.
- André Hostílio Hubert[5];

  Database organization and data collection.
- Elisandréa Sguario Kemper[6];

  Database organization and data collection.

For peer review only - http://bmjopen.bmj.com/site/about/guidelines.xhtml

- Renilson Rehem de Souza[6];

Database organization and data collection.

- Cristiane Feitosa Salviano[6];

Database organization and data collection.

- Hevelin Silveira e Silva[6];

Database organization and data collection.

- Eduardo Barbosa Coelho[7];

Paper writing and data analysis.

- Giuseppe Cesare Gatto[7];

Database organization and data collection.

- Rafael Freitas de Morais[7];

Database organization.

- Leonardo Nunes Alegre[7],

Database organization.

- Rodrigo Citton Padilha dos Reis[8],

This researcher contributed with data collection.

- Joaquim Francisco dos Santos Neto[8],

Database organization and data collection.

- Andresa Fontoura Garbini[1];

This researcher contributed with data collection and analysis.

- Cesar Perdomo Purper[1],

Database organization.

- Veridiana Baldon dos Santos[1];

This researcher contributed with data collection and analysis.

- Rafaela dos Santos Charão de Almeida[1];

This researcher contributed with data collection and analysis.

- Bruna Donida[1];

Database organization and data collection.

For peer review only

- Rogério Farias Bitencourt[1];

Database organization.

- Luciane Kopittke[1];

This researcher contributed with data collection, paper writing and analysis.

- Fernanda Costa dos Santos[1];

This researcher contributed with data collection and analysis.

- Raquel Lutkmeier[1];

Paper writing and data analysis.

- Daniela dos Reis Carazai[1];

This researcher contributed with data collection and analysis.

- Virgínia Angélica Silveira Reis[9];

Database organization and data collection.

- Flávio Clemente Deulefeu[9];

Database review, partial data analysis and data collection.

- Fernanda Gadelha Severino[9];
- This researcher contributed with data collection and analysis.
- José Gustavo da Costa Neto[9];

This researcher contributed with data collection and analysis.

- Nirvania do Vale Carvalho[10],

Database review, partial data analysis and data collection.

- André Jamson Rocha de Andrade[10];

This researcher contributed with data collection and analysis.

- Adriana Melo Teixeira[11];

The author contributed to the organization of the group of researchers.

- Olavo Braga Neto[11],

The author contributed to the organization of the group of researchers.

- Gabriel Cardozo Muller[8],

For peer review only

This researcher contributed with data collection and analysis.

- Ricardo de Souza Kuchenbecker[8]

The author contributed to the organization of the group of researchers, substantial organization of the protocol, improvements in the course of the project, data collection and analysis and writing of the protocol.

For peer review only

Figure 1. Hospitals participating in the survey by size classification

For peer review only

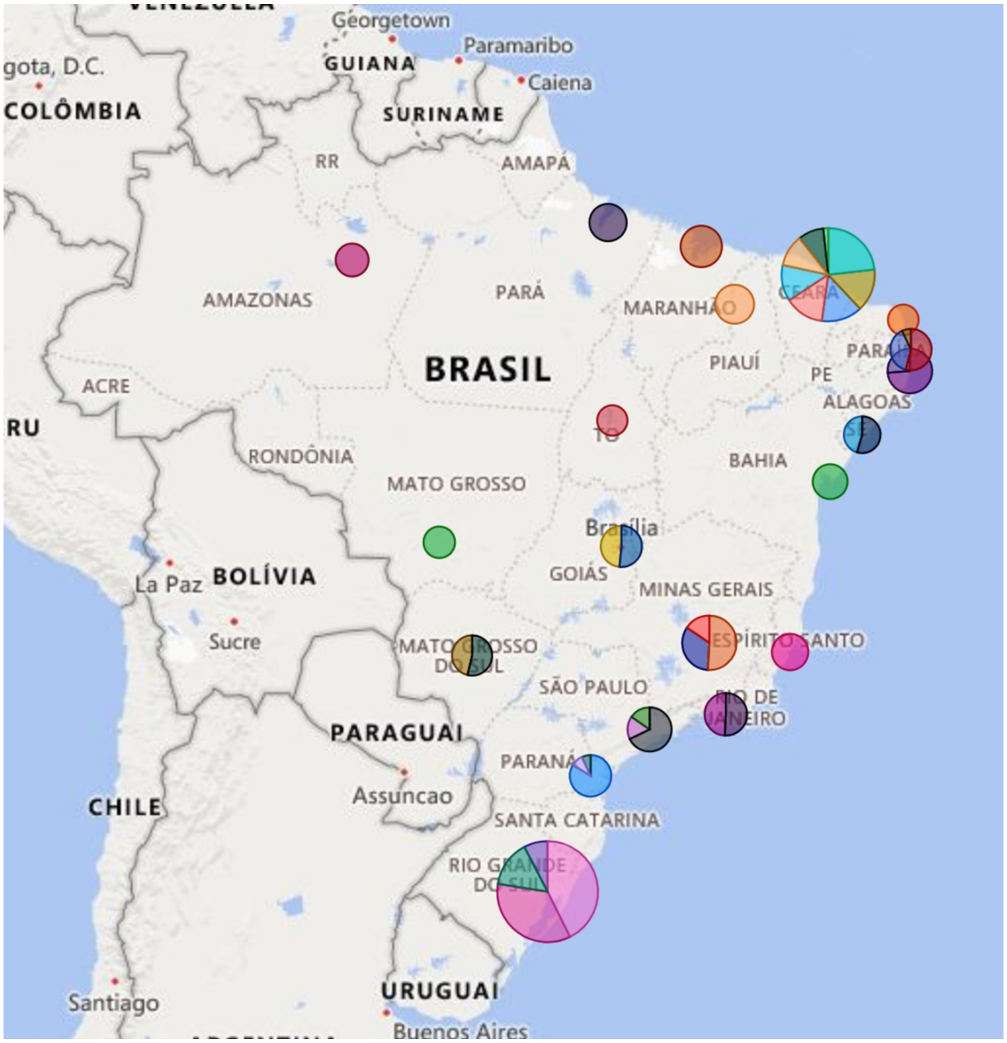

The area of the circle represents the number of beds and the fractions represent the existence of more participating hospitals per state. Source: DATASUS and the Brazilian National Register of Health Service Providers (CNES) (10,21)

Supplementary 1

Description of the analysis objectives

Objective 1. Description of socio-demographic and clinical characteristics

Socio-demographic and clinical characteristics will be described for the population and pre-specified subgroups. Table 3 presents the description of the study variables with their respective descriptive statistics. Tables and graphs will be used to present the results.

For peer review only

Table 3. Study variables and their values or categories and numerical summary

**Variable**	**Numerical summary**
Age (continuous and categorical)<br>< 18 years old<br>0-4 years<br>5-13 years<br>14-17 years<br>18-45 years<br>46-65 years<br>66-75 years<br>> 75 years old	mean (SD) or median (IQR), when continuous and n/N (%), when categorical
Gender<br>Male, female<br>If female, pregnancy status	n/N (%)
Anthropometry and vital signs on	
admission Temperature (continuous and categorical > 37.5ºC)	mean (SD) or median (IQR), when
Heart rate (continuous and categorical > 100 p/ min in adults and adolescents; > 120 in children up to 12 years)	continuous and n/N (%), when categorical mean (SD) or median (IQR), when continuous and n/N (%),
Respiration rate (continuous and categorical > 20 r/min in adults and adolescents; > 40 in children up to 12 years)	when categorical<br>mean (SD) or median (IQR), when continuous and n/N (%), when
Blood pressure (continuous and categorical systolic > 140 mmHg in	categorical
adults) Oxygen saturation (continuous and categorical: <90%, <94%)	mean (SD) or median (IQR), when continuous and n/N (%), when categorical mean (SD) or median (IQR), when continuous and n/N (%), when categorical

For peer review only

Oxygen saturation in room air/oxygen therapy (categorical)	
Glasgow Coma Scale	mean (SD) or median (IQR)
Height (cm)	mean (SD) or median (IQR)
Weight (kg)	mean (SD) or median (IQR)
BMI (continuous: kg/m² and categorical: ≥ 30; ≥ 40)	mean (SD) or median (IQR), when continuous and n/N (%), when categorical
Obesity (BMI ≥ 30)	n/N (%)
Chronic conditions<br>Chronic heart disease (not hypertension); Diabetes; Hypertension; Current smoker; Chronic lung disease; Tuberculosis; Asthma; Asplenia; Chronic kidney disease; Malignant neoplasm; Chronic liver disease (categorical Yes/No); Other (open text field)	n/N (%)
Pre-admission or chronic medications<br>ACE inhibitors; ARBs; NSAIDs; Antiviral: (if yes, which: chloroquine/hydroxychloroquine, azithromycin, kaletra [lopinavir-ritonavir], favipiravir, Other)	n/N (%)
Signs and symptoms on admission and during hospitalization	n/N (%)
Laboratory examinations on admission and during hospitalization<br>Hemoglobin (g/L); Creatinine (mmol/L); White blood cell count (x10g/L); Sodium (mEq/L); Hematocrit (%); Potassium (mEq/L); Platelets (x10g/L); Procalcitonin (ng/mL); APTT/PT; CRP (mg/L); PT (seconds); LDH (U/L); INR; Creatine kinase (U/L); ALT/SGPT (U/L);	mean (SD) or median (IQR)

For peer review only

Troponin (ng/mL)(mEq/L); Total bilirubin (µmol/L); ESR (mm/hr); AST/SGOT (U/L); D-dimer (mg/L); Urea (mmol/L); Ferritin (ng/mL); Lactate (mmol/L)	
Treatments on admission and during hospitalization	n/N (%)
Antiviral (If Yes, which: Ribavirin, Lopinavir/Ritonavir, Neuraminidase Inhibitor, Interferon alpha, Interferon beta, Other; If Other, specify); Corticosteroids (If Yes, route: Oral, Intravenous, Inhaled; If Yes, specify agent; If Yes, provide maximum daily dose); Antibiotic; Antifungal agent; Antimalarial agent (If Yes, specify); Experimental agent (If Yes, specify); NSAID; ACE inhibitors; ARBs; Systemic anticoagulation	
Examination of the pathogen performed during this disease episode	n/N (%)
Influenza virus (If Positive, specify type); Coronavirus (If Positive, specify type; If Other, specify); Other respiratory pathogen (If Positive, specify type); HIV	
Hospitalization in ICU or High Dependency Unit	
Respiratory and intensive care interventions	n/N (%)
Oxygen therapy; Invasive ventilation (any); Extracorporeal support (ECMO); Liver dysfunction; Pronation; Renal replacement therapy (RRT) or dialysis; Inotropics/vasopressors	n/N (%) n/N (%)

For peer review only

Hospital outcome<br>Discharge alive, Hospitalized, Transferred to another unit, Death, Palliative discharge, Unknown	n/N (%)
Complications during hospitalization<br>Shock; Bacteremia; Convulsion; Bleeding; Meningitis/encephalitis; Endocarditis; Anemia; Myocarditis/pericarditis; Cardiac arrhythmia; Acute kidney injury; Cardiac arrest; Pancreatitis; Pneumonia; Liver dysfunction; Bronchiolitis; Cardiomyopathy (If Yes, specify)	n/N (%)
Time-to-event outcomes Symptom onset (day of disease on admission): days between first symptom and admission Length of stay<br>Length of stay in ICU<br>Days from hospital admission to ICU admission<br>Days from hospital admission to transfer or death (reported as total, for survivors and non-survivors)	median (IQR), mean (SD), survival curve (Kaplan-Meier)

For peer review only

Objective 2. Characterization of access profiles and hospital resource utilization patterns

The characteristics signs/symptoms, laboratory tests on admission, presence of chronic diseases, treatments received, and outcomes will be described for the population and pre-specified subgroups.

Objective 3. Association between clinical/socio-demographic characteristics and outcomes

Measures of association, such as *odds ratio* (OR), risk ratio (RR) or *hazard ratio* (HR), crude and adjusted for potential confounding variables will be estimated and 95% confidence intervals (95% CI) will be reported. Logistic regression (or Poisson models with robust variance) will be fitted for dichotomous outcomes. Cox proportional hazards models will be fitted for time-to-event outcomes. The assumptions of the regression models will be evaluated using goodness of fit statistics (likelihood and variance analysis) and residual analysis. The set of adjustment variables will be specified primarily by building theoretical models using causal diagrams (*directed acyclic graphs* - DAGs) [5,6].

Objective 4. Sources of inequities

The distribution of socio-demographic, clinical, and outcome characteristics will be described and compared between pre-specified subgroups. Crude association measures (OR, RR or HR, depending on the nature of the outcome) adjusted for potential confounding variables will be estimated and 95% CIs will be reported. The set of adjustment variables will be primarily specified by building theoretical models (DAGs).

For peer review only

STROBE Statement - **Brazilian network for collaborative research on COVID-19: strengthening WHO global data**

	Item No	Recommendation	Page No
**Title and abstract**	1	(*a*) Indicate the study's design with a commonly used term in the title or the abstract<br>(*b*) Provide in the abstract an informative and balanced summary of what was done and what was found	1, 3
**Introduction**			
Background/rationale	2	Explain the scientific background and rationale for the investigation being reported	5-8
Objectives	3	State specific objectives, including any prespecified hypotheses	9, 12
**Methods**			
Study design	4	Present key elements of study design early in the paper	8-17
Setting	5	Describe the setting, locations, and relevant dates, including periods of recruitment, exposure, follow-up, and data collection	10, 12
Participants	6	(*a*) Give the eligibility criteria, and the sources and methods of selection of participants. Describe methods of follow-up<br>(*b*) For matched studies, give matching criteria and number of exposed and unexposed	10
Variables	7	Clearly define all outcomes, exposures, predictors, potential confounders, and effect modifiers. Give diagnostic criteria, if applicable	10,11, 12 and supplementary data
Data sources/ measurement	8*	For each variable of interest, give sources of data and details of methods of assessment (measurement). Describe comparability of assessment methods if there is more than one group	15 and supplementary data
Bias	9	Describe any efforts to address potential sources of bias	11,14
Study size	10	Explain how the study size was arrived at	10
Quantitative variables	11	Explain how quantitative variables were handled in the analyses. If applicable, describe which groupings were chosen and why	supplementary data
Statistical methods	12	(*a*) Describe all statistical methods, including those used to control for confounding<br>(*b*) Describe any methods used to examine subgroups and interactions<br>(*c*) Explain how missing data were addressed<br>(*d*) If applicable, explain how loss to follow-up was addressed<br>(*e*) Describe any sensitivity analyses	11
**Results**			
Participants	13*	(a) Report numbers of individuals at each stage of study—eg numbers potentially eligible, examined for eligibility, confirmed eligible, included in the study, completing follow-up, and analysed<br>(b) Give reasons for non-participation at each stage<br>(c) Consider use of a flow diagram	17
Descriptive data	14*	(a) Give characteristics of study participants (eg demographic, clinical, social) and information on exposures and potential confounders<br>(b) Indicate number of participants with missing data for each variable of interest<br>(c) Summarise follow-up time (eg, average and total amount)	17
Outcome data	15*	Report numbers of outcome events or summary measures over time	17

For peer review only - http://bmjopen.bmj.com/site/about/guidelines.xhtml

Main results	16	(*a*) Give unadjusted estimates and, if applicable, confounder-adjusted estimates and their precision (eg, 95% confidence interval). Make clear which confounders were adjusted for and why they were included<br>(*b*) Report category boundaries when continuous variables were categorized<br>(*c*) If relevant, consider translating estimates of relative risk into absolute risk for a meaningful time period	
Other analyses	17	Report other analyses done—eg analyses of subgroups and interactions, and sensitivity analyses	15,16
**Discussion**			
Key results	18	Summarise key results with reference to study objectives	21
Limitations	19	Discuss limitations of the study, taking into account sources of potential bias or imprecision. Discuss both direction and magnitude of any potential bias	18
Interpretation	20	Give a cautious overall interpretation of results considering objectives, limitations, multiplicity of analyses, results from similar studies, and other relevant evidence	19,20
Generalisability	21	Discuss the generalisability (external validity) of the study results	19,20
**Other information**			
Funding	22	Give the source of funding and the role of the funders for the present study and, if applicable, for the original study on which the present article is based	17

*Give information separately for exposed and unexposed groups.

Note: An Explanation and Elaboration article discusses each checklist item and gives methodological background and published examples of transparent reporting. The STROBE checklist is best used in conjunction with this article (freely available on the Web sites of PLoS Medicine at http://www.plosmedicine.org/, Annals of Internal Medicine at http://www.annals.org/, and Epidemiology at http://www.epidem.com/). Information on the STROBE Initiative is available at http://www.strobe-statement.org.

For peer review only

BMJ Open

Cohort Study Protocol of the Brazilian Collaborative Research Network on COVID-19: strengthening WHO global data

Journal:	*BMJ Open*
Manuscript ID	bmjopen-2022-062169.R1
Article Type:	Protocol
Date Submitted by the Author:	16-Aug-2022
Complete List of Authors:	Anschau, Fernando; Grupo Hospitalar Conceição, Research Sector; Universidade Federal do Rio Grande do Sul, Programa de Pós Graduação em Neurociências - ICBS<br>Aredes, Natália; Universidade Federal de Goiás, Nursing School; Brazilian Company of Hospital Services<br>Reveiz, Ludovic; Pan American Health Organization (PAHO) - World Health Organization (WHO)<br>Padilla, Monica; Pan American Health Organization Brazil<br>Gomes, Rosane ; Pan American Health Organization Brazil<br>Carvalho , Wellington ; Pan American Health Organization Brazil<br>Leles, Fernando ; Pan American Health Organization Brazil<br>Reese, Fernanda; Complexo Hospitalar do Trabalhador<br>Hubert, André ; Complexo Hospitalar do Trabalhador<br>Kemper, Elisandréa ; Hospital da Criança de Brasília<br>de Souza , Renilson; Hospital da Criança de Brasília<br>Salviano, Cristiane ; Hospital da Criança de Brasília<br>e Silva, Hevelin ; Hospital da Criança de Brasília<br>Coelho, Eduardo; Brazilian Company of Hospital Services<br>Gatto, Giuseppe ; Brazilian Company of Hospital Services<br>de Morais, Rafael; Brazilian Company of Hospital Services<br>Alegre, Leonardo; Brazilian Company of Hospital Services<br>Padilha dos Reis, Rodrigo ; Universidade Federal do Rio Grande do Sul, Graduate Program in Epidemiology ; Hospital de Clínicas de Porto Alegre<br>dos Santos Neto , Joaquim; Universidade Federal do Rio Grande do Sul, Graduate Program in Epidemiology; Hospital de Clínicas de Porto Alegre<br>Garbini, Andresa; Grupo Hospitalar Conceição<br>Purper, César; Grupo Hospitalar Conceicao Gerencia de Ensino e Pesquisa<br>dos Santos, Veridiana ; Grupo Hospitalar Conceição<br>Charão de Almeida, Rafaela; Grupo Hospitalar Conceição<br>Donida, Bruna; Grupo Hospitalar Conceicao Gerencia de Ensino e Pesquisa<br>Bitencourt, Rogério; Grupo Hospitalar Conceição Gerência de Ensino e Pesquisa<br>Kopittke, Luciane; Grupo Hospitalar Conceição Gerência de Ensino e Pesquisa<br>dos Santos, Fernanda; Grupo Hospitalar Conceição<br>Lutkmeier, Raquel; Grupo Hospitalar Conceição<br>Carazai, Daniela ; Grupo Hospitalar Conceição

	Reis , Virgínia ; Instituto de Saúde e Gestão Hospitalar<br>Deulefeu, Flávio ; Instituto de Saúde e Gestão Hospitalar<br>Severino, Fernanda; Instituto de Saúde e Gestão Hospitalar<br>da Costa Neto, José Gustavo ; Instituto de Saúde e Gestão Hospitalar<br>Carvalho, Nirvania ; Health Department of the State of Piauí, Hospital Getúlio Vargas<br>de Andrade, André ; Health Department of the State of Piauí, Hospital Getúlio Vargas<br>Teixeira, Adriana; Brazilian Ministry of Health, Department of Hospital, Home, and Emergency Care (DAHU) of the Specialized Health Care Office (SAES)<br>Braga Neto, Olavo ; Brazilian Ministry of Health, Department of Hospital, Home, and Emergency Care (DAHU) of the Specialized Health Care Office (SAES)<br>Muller, Gabriel ; Universidade Federal do Rio Grande do Sul, Graduate Program in Epidemiology; Hospital de Clínicas de Porto Alegre<br>Kuchenbecker, Ricardo; Universidade Federal do Rio Grande do Sul, Graduate Program in Epidemiology ; Hospital de Clínicas de Porto Alegre
**Primary Subject Heading**:	Epidemiology
Secondary Subject Heading:	Health services research
Keywords:	COVID-19, EPIDEMIOLOGY, PUBLIC HEALTH

SCHOLARONE™
Manuscripts

Cohort Study Protocol of the Brazilian Collaborative Research Network on COVID-19: strengthening WHO global data

Authors:

Fernando Anschau[1], Natália Del' Angelo Aredes[2], Ludovic Reveiz[3]; Monica Padilla[3]; Rosane de Mendonça Gomes[4]; Wellington Mendes Carvalho[4]; Fernando Antonio Gomes Leles[4]; Fernanda Baeumle Reese[5]; André Hostílio Hubert[5]; Elisandréa Sguario Kemper[6]; Renilson Rehem de Souza[6]; Cristiane Feitosa Salviano[6]; Hevelin Silveira e Silva[6]; Eduardo Barbosa Coelho[7]; Giuseppe Cesare Gatto[7]; Rafael Freitas de Morais[7]; Leonardo Nunes Alegre[7], Rodrigo Citton Padilha dos Reis[8], Joaquim Francisco dos Santos Neto[8], Andresa Fontoura Garbini[1]; Cesar Perdomo Purper[1], Veridiana Baldon dos Santos[1]; Rafaela dos Santos Charão de Almeida[1]; Bruna Donida[1]; Rogério Farias Bitencourt[1]; Luciane Kopittke[1]; Fernanda Costa dos Santos[1]; Raquel Lutkmeier[1]; Daniela dos Reis Carazai[1]; Virgínia Angélica Silveira Reis[9]; Flávio Clemente Deulefeu[9]; Fernanda Gadelha Severino[9]; José Gustavo da Costa Neto[9]; Nirvania do Vale Carvalho[10], André Jamson Rocha de Andrade[10]; Adriana Melo Teixeira[11]; Olavo Braga Neto[11], Gabriel Cardozo Muller[8], Ricardo de Souza Kuchenbecker[8]

[1] Hospital Nossa Senhora da Conceição – Grupo Hospitalar Conceição

[2] Nursing School, Universidade Federal de Goiás, member of the EBSERH research team

[3] Pan American Health Organization (PAHO) - World Health Organization (WHO)/Washington, DC, US

[4] PAHO/WHO in Brazil

[5] Complexo Hospitalar do Trabalhador

[6] Hospital da Criança de Brasília

[7] Brazilian Company for Hospital Services (Empresa Brasileira de Serviços Hospitalares - EBSERH)

[8] Graduate Program in Epidemiology UFRGS, Hospital de Clínicas de Porto Alegre

[9] Instituto de Saúde e Gestão Hospitalar

[10] Hospital Getúlio Vargas- Teresina, Piauí - Health Department of the State of Piauí

[11] Department of Hospital, Home, and Emergency Care (DAHU) of the Specialized Health Care Office (SAES) of the Brazilian Ministry of Health

For peer review only

Cohort Study Protocol of the Brazilian Collaborative Research Network on COVID-19: strengthening WHO global data

Corresponding author:

Fernando Anschau
Av. Francisco Trein n.:326
Bairro Cristo Redentor – Porto Alegre, Rio Grande do Sul,
Brazil Zip Code: 91350-200
e-mail: afernando@ghc.com.br
telefone number: +55 (51) 3357 2805

Word Count: 4975

For peer review only

Cohort Study Protocol of the Brazilian Collaborative Research Network on COVID-19: strengthening WHO global data

Abstract

Introduction: With the COVID-19 pandemic, hospitals in low-income countries were faced with a triple challenge. First, a large number of patients required hospitalization because of the infection's more severe symptoms. Second, there was a lack of systematic and broad testing policies for early identification of cases. Third, there were weaknesses in the integration of information systems, which led to the need to search for available information from the hospital information systems. Accordingly, it is also important to state that relevant aspects of COVID-19's natural history had not yet been fully clarified. The aim of this research protocol is to present the strategies of a Brazilian network of hospitals to perform systematized data collection on COVID-19 through the World Health Organization (WHO) Platform. **Methods and Analysis:** This is a multicenter project among Brazilian hospitals to provide data on COVID-19 through the WHO global platform, which integrates patient care information from different countries. From October 2020 to March 2021, a committee worked on defining a flowchart for this platform, specifying the variables of interest, data extraction standardization and analysis. **Ethics and Dissemination:** This protocol was approved by the Research Ethics Committee (CEP) of the Research Coordinating Center of Brazil (CEP of the Hospital Nossa Senhora da Conceicao), on January 29, 2021, under approval No. 4.515.519 and by the National Research Ethics Commission (CONEP), on February 5, 2021, under approval No. 4.526.456. The project results will be explained in WHO reports and published in international peer-reviewed journals, and summaries will be provided to the funders of the study.

Keywords: COVID-19, clinical data, morbidity, mortality

Strengths and limitations of this study:

Strengths:

- The information generated by this research, allows the development of maps of the evolution of SARS-CoV-2 infection;
- This research collected information, which enables the development of public policies to face pandemics.
- This research generated data covering different moments of the COVID-19 pandemic; it has data obtained in the SARS-COV pre-vaccination era and after the vaccines.

Limitations:

- This research is an observational study;
- This research involves a convenience and non-probabilistic sample of hospitalized patients, and then it may not represent the population of hospitalized patients with COVID-19.

For peer review only

Cohort Study Protocol of the Brazilian Collaborative Research Network on COVID-19: strengthening WHO global data

Introduction

COVID-19 is a disease caused by SARS-CoV-2, which started in Wuhan (Hubei province, China) in December 2019 (1), and it was characterized as a pandemic by the World Health Organization (WHO) on March 11, 2020 (2). The disease caused by SARS-CoV-2 has a lower lethality when compared to that of two other coronaviruses that have already caused epidemics, namely SARS-CoV and MERS-CoV. However, its high transmission rate places a great demand on health systems, with an increase in the number of hospitalizations, need for oxygen by assisted ventilation, and other forms of clinical support (3-5).

Brazil ranks third worldwide in the total number of COVID-19 cases, with all regions affected (6,7), and demographic and social inequalities impacted the results obtained in transmission control and health care. Low- and middle-income countries, such as Brazil, had great difficulty adopting pandemic mitigating measures such as non-pharmacological interventions (8). Along with the high transmissibility of variants of interest, so-called *variants of concern* (VOC), the pandemic's increased extent was understandable (9,10).

Brazil is a country of continental dimensions, with 6,820 hospitals, of which 2,423 are public hospitals and 4,397 are private hospitals (11). The country has 536,474 hospital beds (1.96 and 0.8 beds per 1,000 inhabitants in private and public hospitals, respectively) (11,12). There are disparities in the availability of hospital beds in the different regions, including those in the intensive care unit (ICU), which is of utmost importance for the care of COVID-19 patients. For every 100,000 inhabitants, there are 9.73 ICU beds in the Unified Health System (Sistema Único de Saúde - SUS) in the North region, 10.46 in the Northeast region, 11.87 in the Central-West region, 13.24 in the Southeast region, and 15.88 in the South region, according to data from 2020 (13).

For peer review only - http://bmjopen.bmj.com/site/about/guidelines.xhtml

Between December 2019 and April 2020, ICU beds were expanded from 46,045 to 60,265 (13) nationwide with an overall increase of 30.88%, with the increase in ICU beds under SUS being almost four times smaller than the increase in ICU beds in the private network (13.46% increase under SUS and 48.33% in the private sector), signaling a need for public investment, especially in view of the gaps identified during the pandemic.

A study that evaluated the first 250,000 SARS-Cov-2 hospitalizations in Brazil from the national Severe Acute Respiratory Syndrome case database (SIVEP-Gripe) highlighted the in-hospital mortality rate, which reached 38%, the number of people with demands compatible with the ICU (39%) and regional discrepancies, revealing considerable challenges in managing the pandemic in Brazil (14). However, the results showed a high proportion of missing data for some variables, especially comorbidities and symptoms, and non-standardized reporting protocols (14). Additionally, these data did not include the so-called "second wave", caused predominantly in Brazil by new highly transmissible variants such as P1 and B1.1.7 (15-17).

Data from the two waves observed in Brazil using the same dataset (SIVEP-Gripe) were published in 2021. The cross-sectional study of hospitalized patients with RT-PCR positive for SARS-CoV-2 in Brazil comprised data entered into SIVEP-Gripe from February 25, 2020 to April 30, 2021, separated into two waves on November 5, 2020. As opposed to what was seen in the rest of the world, in-hospital mortality in Brazil increased from 34.81% in the first wave to 39.30% in the second wave. In the second wave, there were more ICU admissions, use of non-invasive and invasive ventilation, and increased mortality in the younger age groups. The Brazilian South and Southeast regions had the highest hospitalization rates per 100,000 inhabitants. Nevertheless, the in-hospital mortality rate was higher in the northern and northeastern states. Racial differences were observed in clinical outcomes, with white people being the most hospitalized, though black/brown-skinned people had higher mortality rates.

For peer review only

The younger age groups displayed more considerable differences in mortality compared to the groups with and without comorbidities in both waves. The results reflect the inequalities observed in Brazil and the overload of health systems upon the second wave's arrival (18).

It is critical to maintain surveillance on the pandemic's evolution over time and across the country to measure its impact on the health care system. In Brazil, SUS consists of a public network of services at all levels of care, on which about 75% of the population depends exclusively. Today, 66.34% of hospital beds belong to SUS (19), which characterizes the Brazilian public system as responsible for most of the health care during the COVID-19 pandemic in the country. Even so, both public and private hospitals do not use a unified electronic record system, which increases the heterogeneity of reports and represents an obstacle for data collection in research.

As a strategy to overcome the challenges of data collection for health research and management purposes, the Pan American Health Organization (PAHO)-WHO proposed a collaboration in 2020 between hospitals in all regions of Brazil to join databases and collect clinical data from COVID-19 patients. Through this initiative, it was possible to gather data from different locations around the country, and the world, to characterize the disease's natural history, identify risk factors for severe cases and describe treatment interventions and outcomes. Thus, it directly contributes to national and global public health with scientific evidence on COVID-19 clinical management and the strengthening of research networks and technology-supported data collection.

As far as we know, this is the first initiative in Brazil to create a "Big Data" database in health care from hospitals and, afterwards, a *data hub* with clinical conditions and usage patterns from the health care system.

The aim of this study is to provide standardized quality clinical data on the COVID-19 pandemic in Brazil. Furthermore, it aims to describe the resources and methods used in this collaboration to identify and validate the cases in each database, with patients confirmed as infected with SARS-CoV-2 by laboratory tests (case group)

For peer review only

and suspects (control group).

Methods

This study is being conducted in 43 hospitals located in all regions of Brazil. From March 2020 to June 2022, a committee of experts worked on defining a blueprint for this platform, specifying variables of interest, data extraction standardization and data analysis.

Aiming to standardize data collection, the project uses *case report forms* (CRF) to extract clinical information from adults and children hospitalized with COVID-19 (accessed from the platform URL: https://www.who.int/teams/health-care-prontidão-clínica-unidade/COVID-19/data platform). The data from all the centers surveyed can be gathered and analyzed - either together or by center or region of the country - to provide a better understanding of the disease and determine the public health response to the pandemic. Collecting anonymized clinical data globally will provide WHO the following:

1. Identification of the main clinical characteristics and prognostic factors of hospitalization cases for suspected or confirmed COVID-19, expanding our knowledge of the severity, spectrum and impact of the disease on the hospitalized population globally and in different countries.
2. Identification of clinical interventions, facilitating global and country operational planning during the COVID-19 pandemic.

The research questions and hypotheses of the Brazilian protocol of the Global Clinical Platform on COVID-19 are presented in Table 1 below.

Table 1 - Project research questions and hypotheses

Questions	Hypotheses

For peer review only - http://bmjopen.bmj.com/site/about/guidelines.xhtml

Are the descriptions presented in the international literature regarding infections by the SARS-COV-2 applicable to the Brazilian reality?	The descriptions presented in the existing academic literature on the clinical, laboratory, radiological and therapeutic profile of patients diagnosed with the SARS-COV-2 and admitted to Brazilian hospitals are different, a factor that makes it essential to consider Brazilian peculiarities during this disease's screening, diagnosis and treatment.
What are the main symptoms presented by Brazilian patients with COVID-19, especially those admitted to different hospital units in Brazil?	The most common clinical manifestations in Brazilian patients tested for SARS-COV-2 are related to fever, myalgia, respiratory symptoms, and headache.
Is in-hospital mortality from COVID-19 similar in Brazil and the rest of the world?	The in-hospital mortality profile of patients admitted to Brazilian hospitals with COVID-19 is not similar to that presented in the literature.

1

For peer review only

The primary and secondary objectives of the Brazilian protocol of the Global Clinical Platform on COVID-19 are as follows:

- Primary objective: to determine the clinical, laboratory and radiological profile, therapy and mortality of patients confirmed having COVID-19 infection and admitted to Brazilian hospitals.
- Secondary objectives: 1) to identify outcomes such as ICU admission, length of hospital stay and duration of mechanical ventilation and of respiratory therapy; 2) to assess the incidence of adult respiratory dysfunction syndrome and need for mechanical ventilation and intensive care, and days of intensive care in patients admitted to hospitals; and 3) to identify the incidence of signs and symptoms of multisystemic inflammatory syndrome and associated chronic diseases.

Study design, population and sample

This is a multicenter cohort study with convenience sampling from the population of patients hospitalized with COVID-19, because of either clinical suspicion or laboratory confirmation. As inclusion criteria for cases, patients with confirmed COVID-19 (RT-PCR tests or rapid tests), admitted to hospitals with a minimum length of stay of 24 hours, are considered. Patients admitted to one of the hospital units who tested positive for COVID-19 in the period from March 2020 to June 2022 will be entered in the study retrospectively.

Main outcomes and parameters

The CRFs were developed by WHO for the collection and submission of harmonized data to the COVID-19 Global Clinical Platform, and the procedures for further data collection were performed by the committee's Technical Team.

Key descriptive parameters include socio-demographic data (age and sex), presence of preexisting conditions, use of chronic medications, clinical characteristics on admission and during hospitalization, laboratory findings on admission and during

For peer review only

For peer review only - http://bmjopen.bmj.com/site/about/guidelines.xhtml

hospitalization, clinical interventions on admission and during hospitalization (use of oxygen, use of mechanical ventilation, use of therapeutics), and patient outcomes (death, discharge and referral).

The patient outcomes described above will be used in secondary analysis to investigate associations between baseline characteristics and disease severity and outcomes.

Statistical considerations

General considerations

Statistical analyses (numerical summaries, point estimates, confidence intervals, hypothesis tests, fitting regression models, tables, graphs and maps) will be performed in R[3] version 4.0.5 or higher. Specification of sets of adjustment variables in certain association analyses will be conducted in DAGitty[4] software.

Missing data

For each analysis, the denominator will represent the number of available observations.

The date of hospital admission will be considered essential and critical to know the hospitalized population to be analyzed. Records with missing admission dates will be excluded from the analysis unless there is confirmation from participating hospitals that the data refer to a cohort of hospitalized patients.

Data imputation will not be performed but can be considered for each particular analysis.

Descriptive statistics

According to the type of variable, the descriptive statistics will be as follows:

- Quantitative variables: number of observations (N), mean, standard deviation (SD), median and interquartile range (IQR), depending on the distribution of the variable.

For peer review only - http://bmjopen.bmj.com/site/about/guidelines.xhtml

- Qualitative variables: number of observations (N), absolute frequency (n), and relative frequency (%).
- Percentages will be calculated for the number of participants with observed data (n/N).

Objectives of the analysis (See Supplementary 1 table 1)

1. Description of socio-demographic and clinical characteristics

To describe the socio-demographic and clinical characteristics of hospital admissions, interventions and clinical outcomes among:

- people infected with the SARS-CoV-2 virus cared for by hospitals within the Global Platform;
- specific population groups, such as children, pregnant women and populations with co-infections or comorbidities, among others.

2. Characterization of access profiles and hospital resource utilization patterns

To describe access profiles and hospital resource utilization patterns such as emergency care, inpatient admissions in wards and ICU, including non-invasive and invasive ventilation devices and non-traditional care modalities such as intermediate units, ventilatory support and others.

3. Association between clinical/socio-demographic characteristics and outcomes To I

To investigate factors associated with disease severity, ICU admission and in-hospital mortality among the following:

- people infected with the SARS-CoV-2 virus cared for by hospitals within the Global Platform;
- specific population groups, as described above.

4. Sources of inequities

To investigate potential sources of inequities in timely access to diagnostic and therapeutic services and resources and the association with clinical outcomes.

For peer review only - http://bmjopen.bmj.com/site/about/guidelines.xhtml

Data governance

Defining data governance is always necessary in a research protocol, considering that data is protected by legislation, including the rights of research participants. This model dictates that the hospitals involved understand the research aspects, the information security points, and complete the process of sending data to the central repository in a clear, unambiguous manner. Furthermore, regarding information security, the central repository's teams are required to follow procedures ensuring ethical and secure actions, in compliance with the legislation in force, when processing information.

Privacy and Consent Policies are important points for discussion on health data sharing. Therefore, for the preparation of this proposal, the concepts and technical principles of the General Data Protection Act (LGPD) were respected, considering health data as sensitive, as described in these documents (20). The LGPD regulates the processing of personal data and aims to protect citizens' right to freedom, privacy and free development.

Considering all these aspects, three levels of governance/access were defined: strategic, tactical and operational:

1. The Advisory Committee of the PAHO/WHO COVID-19 Platform Project represents the first, whose main objective is to technically support PAHO/WHO in the terms of the COVID-19 Global Clinical Data Platform, and the participating institutions in the activities of preparation for analysis and publication of clinical and epidemiological data;
2. The second consists of those responsible for translating the strategy and integrating all parties, setting the objectives and managing the project, including defining the work processes, schedule, team engagement and communication, quality, and results follow-up;
3. The third performs activities from the tactical level always in compliance with data

For peer review only - http://bmjopen.bmj.com/site/about/guidelines.xhtml

security standards and rules. It is at this level that statements like "data must be stored securely" translate into technological tools ensuring the semantics read into the sentence.

Risks

Risks are related to the loss of confidentiality. However, they have been and will be constantly mitigated/controlled by the anonymization of the participants' identification data. The researchers take responsibility for maintaining their commitment to preserve data security and confidentiality, and hospital institutions are asked to take all necessary steps to protect their access credentials and passwords to the COVID-19 Data Platform.

The collected and anonymized data will be stored by the aforementioned WHO Platform, a secure, password-protected, restricted-access electronic platform. WHO commits to the following: i) to protect the confidentiality of anonymous data on COVID-19 and to prevent its unauthorized disclosure; ii) to implement and maintain technical and organizational security measures to protect the security of anonymous data on the Platform; and iii) to follow the International Health Regulations (21).

Benefits

It is expected that this protocol will produce specific and reliable knowledge of the COVID-19 pandemic - clinical and epidemiological analysis, leading to discussions on Brazil and other countries and their reality and plans to fight the pandemic. It is expected that it will deepen our knowledge of the pandemic scenario and help hospital institutions to develop preventive measures and health service protocols and to strengthen staff training in existing complications. The information generated by the research can serve as a basis for developing maps of the SARS-CoV-2 infection evolution.

Clinical data extraction

As the WHO representative in the country, the PAHO/WHO office in Brazil,

For peer review only - http://bmjopen.bmj.com/site/about/guidelines.xhtml

through the Technical Unit of Health Systems and Services, coordinated the project by dialoguing with the partner institutions to make this project effective. In this context, Grupo Hospitalar Conceição (GHC) became the national coordinating Brazilian institution for submission procedures to the Research Ethics Committee (CEP) and the National Research Ethics Commission (CONEP).

Data collection started retrospectively in 43 Brazilian hospitals - as shown in Figure 1 - starting from the first COVID-19 case diagnosed in Brazil (March 2020) and ending in June 2022. We chose to carry out this first stage of data collection directly in the hospital units, consulting the databases of the clinical and care information systems available in each institution or the physical records of the hospitalizations of patients with suspected or confirmed COVID-19 in these units. Figure 2 shows the flowchart of the study protocol.

The selected hospitals are responsible for providing all the conditions for the project's execution, such as access to data collection and feeding the platform to the PAHO/WHO consultant assigned to each institution. Accordingly, each hospital signed a commitment form that contains all the guarantees offered by the WHO to maintain data confidentiality and anonymization. The collection and access process, in agreement with each hospital unit, respects the specificities and internal regulations, and also follows the ethical and data security norms.

Considering that the information contained in the hospital records of each institution can be divided between structured and open text, and in view of the heterogeneities of Brazilian hospitals in terms of management, there is a need to organize data collection in several ways: one through manual consultation of electronic citizen records (ECR) - or physical records for hospitals that do not have ECRs - and filling out the WHO-specific forms; another through the development of an automated programming interface (API) for filling out these forms.

The standardized API was developed by information technology (IT) professionals to share databases from different hospitals. These databases, with

For peer review only

provision for secure cloud-based storage, contain de-identified electronic health records (EHRs) corresponding to admission notes for the first 48 hours of care and the 24 hours prior to discharge/outcome for each research participant.

The structured ECR data contains demographic information, prescriptions, laboratory results, vital signs and general patient characterization such as height and weight and admission and outcome dates. Since the lack of data for different variables can be significant, there is the possibility for institutions to search and fill in part of the variables from unstructured data by open text extraction.

For this procedure, the Smart Health Connect (SHC) software is used, which operates on the basis of an algorithm using Deep Neural Networks, extracting information from the clinical evolutions and transferring to the CRF data that were previously missing, reinforcing the completeness of the database exported to the WHO. The main variables of interest incorporated through this software were comorbidities, symptoms and clinical events, especially those related to COVID-19, such as oxygen support and ventilation type, and outcomes. The software is capable of recognizing and categorizing data regarding history (past), current events (present) and expected actions or outcomes (future) and identifying variables related to drug treatment and clinical condition, including vital signs, symptoms, clinical devices, affections/pathologies, and other relevant data that could not be captured in the structured ECR data fields.

SHC uses the newest natural language processing techniques available to the scientific community and a software architecture model that enables mass data extraction.

Regarding the technological standard used by the software, there are two approaches supported by artificial intelligence. The first is Named Entity Recognition (NER), which has the function of locating previously defined entities in open text. For this project, the categories are disease names, signs and symptoms, procedures,

For peer review only

medications, ventilatory support and test results. SHC identifies and marks in the text each of the identified categories in a process known as inference.

Once the relevant terms are identified, the other step is the analysis of the findings in a given text, called Assertion Detection. Through this approach it is possible to differentiate the status of what has or has not actually occurred with that patient. This is necessary because the medical records can indicate something related to the past, or to a third party, such as family history, or even state that the patient does not have a certain condition or does not use a certain medication.

Expected results

At the end of the project, in addition to the reports produced by WHO with the data provided globally and the ensured access to the databases, PAHO/WHO in Brazil will organize publications in partnership with the institutions participating in the project in the country, highlighting the results and Brazil's contribution in fighting the COVID-19 pandemic.

Based on this protocol design, it will be possible to continue data collection among the participating hospitals and continuously add valuable information for public health in the context of facing the pandemic in Brazil. Once collaboration between institutions has taken place and the form of data collection, variables of interest and technologies to support the search for information of interest have been standardized, there is significant potential to advance the analysis of evidence and response measures in the national health system.

Until now, the identification of this strategy's partner hospitals and their characterization, with number and type of beds, provide an analysis perspective of the health care network scenario in the fight against the pandemic (Table 2).

Ethics

This protocol was approved by the Research Ethics Committee (CEP) of the Research Coordinating Center of Brazil (CEP of the Hospital Nossa Senhora da

For peer review only - http://bmjopen.bmj.com/site/about/guidelines.xhtml

Conceição), on January 29, 2021, under approval No. 4.515.519 and by the National Research Ethics Commission (CONEP), on February 5, 2021, under approval No. 4.526.456. Afterwards, it received the approval of the local CEPs of each institution involved. In addition, this study follows the resolutions 466/12 and 510/16 and the resolutions CNS No. 466/2012 and No. 510/2016 of the National Health Council, which deal with the ethical aspects involving the use of data directly obtained from participants or identifiable information, or that may entail risks greater than those existing in everyday life. The results of this study will be published in international peer-reviewed journals and summaries will be provided to the funders of the study.

Strengths and Limitations

Through this initiative it is possible to gather data from different locations around the country to characterize the disease's natural history, identify risk factors for severe cases and describe treatment interventions and outcomes. Thus, it directly contributes to national and global public health with scientific evidence on COVID-19 clinical management and to the strengthening of research networks and technology-supported data collection.

As far as we know, this is the first initiative in Brazil to create a "Big Data" database in health care from hospitals and, afterwards, a *data hub* with clinical conditions and usage patterns from the health care system.

Because this is a convenience, non-probabilistic sample of patients admitted to health care facilities, it may not represent the population of COVID-19 patients hospitalized in all the country. However, the information generated by this research can serve as a basis for the development of maps of the evolution of SARS-CoV-2 infection and public policies to face pandemics.

Patient and public involvement

This project is already involving both Brazilian communities and public agencies that are facing the fight against the COVID-19 pandemic, as well as the Brazilian

For peer review only - http://bmjopen.bmj.com/site/about/guidelines.xhtml

Ministry of Health and PAHO/WHO. We intend to expand partnerships and involve more stakeholders from Brazil.

Discussion

One of the main concerns regarding the COVID-19 pandemic is its burden on the organization of the health care system (22). The need for a general and effective organization of health services to treat the population safely and fight the disease's complications has required a great deal of effort. This includes reallocating health care workers, using appropriate medicines, producing, distributing and delivering vaccines to the population, and increasing health care capacity (23).

The outbreak of the highly transmissible delta variant (B.1.617.2) raises new questions among policy makers and health system managers regarding the impact on hospitalization rates, a point reinforced by a recent published study that indicates a possible worst-case scenario compared to the alpha variant (B.1.1.7) (24). Additionally, according to Twohig KA et al. (2021), this recent research adds to previous evidence and reinforces the notion that the delta variant, in unvaccinated populations, may increase the burden on the overall health care system when compared to the SARS-CoV-2 alpha variant (24).

The Brazilian ICU occupancy condition became critical in most of the country in March 2021, when 20 states reached critical rates (equal to or greater than 80% occupancy) and became intermediate in most states by June 2021 (between 60 and 80%), in concurrence with high SARS-CoV-2 transmission rates (25,26).

It is necessary to maintain biosafety education for the general population (social distancing and the use of masks), encourage them to seek the vaccine when called upon by age/group and complete the recommended number of doses. At the organizational level of hospitals, it is critical to measure resources and indicators, planning strategies according to the literature and lessons learned during the pandemic to meet the next demand. For the public policy level, it is crucial to intensify

For peer review only - http://bmjopen.bmj.com/site/about/guidelines.xhtml

vaccination throughout the country and support the health system by consolidating its structure and strategies for serving the population.

The initial ignorance regarding the virus and the associated disease led hospitals and health services to seek different internal management strategies to meet the demand coming with COVID-19 (27). Berwick DM used the term "choices for the new normal" to point to six properties of care for lasting change: time, standards, working conditions, proximity, readiness and equity. For the author, the changes in care required by the pandemic may allow health systems to become more patient-centered and efficient and consequently provide better care to the public (28).

Since the spectrum of SARS-CoV-2 infection can range from asymptomatic infection to potentially fatal complications of COVID-19, we chose to list patients with suspected and confirmed infection who were admitted to hospital units (29). Thus, we believe that the strategy of the "COVID-19 Global Clinical Data Platform for clinical characterization and management of hospitalized patients with suspected or confirmed COVID-19" contributes to the understanding of the Brazilian context of pandemic crisis in the health care system by providing data analysis of clinical aspects and epidemiology.

It also reinforces a portrait of the reality of the institutions based on the current demand, discussion on aspects of the main organizational frameworks for fighting the pandemic and how to keep the institutions functioning and meeting the health needs of the population amidst the challenges.

For peer review only

Conclusions

This project describes the collaboration of several Brazilian hospitals to build a joint database, with special attention to protocols aimed at reducing heterogeneity between different platforms and ensuring the quality of research data.

As part of the process, the project management carried out an organized multicenter research framework involving PAHO/WHO, as the center of the strategy, and Brazilian public hospitals, resulting in substantial data collection. In addition, the data extraction used innovative techniques in the technology area, promoting quality and mitigating the lack of relevant information, moving towards data collection in unstructured fields, such as clinical evolution which are the texts with the most detailed health data in the medical records.

This proposal accomplishes an important step towards fighting COVID-19 in a developing country of continental dimensions such as Brazil, by building a foundation for new collaborations between referral hospitals and demonstrating the feasibility of joint initiatives for the emergence of "Big Data" analysis strategies.

Acknowledgements

This project received the institutional support from the hospital institutions mentioned here through access to data and research participants, which were critical for this research, the reason for our gratitude.

The authors would like to thank the research participants who made the development of the research project possible.

Author Statement:

A number of people were involved in the development of the research proposal. Fernando Anschau, Bruna Donida, Ricardo de Souza Kuchenbecker, Luciane Kopittke contributed to the organization of the group of researchers, data collection and analysis and writing of the protocol. Natália Del' Angelo Aredes made substantial contributions to the design of the work, data collection based on the protocol (in progress),

For peer review only - http://bmjopen.bmj.com/site/about/guidelines.xhtml

literature review and writing. She also revised the manuscript and approved the submitted version. Ludovic Reveiz, Monica Padilla, Rosane de Mendonça Gomes, Wellington Mendes Carvalho and Fernando Antonio Gomes Leles worked on the organization of the group of researchers and logistical support. Fernanda Baeumle Reese, André Hostílio Hubert, Elisandréa Sguario Kemper, Renilson Rehem de Souza, Cristiane Feitosa Salviano, Hevelin Silveira e Silva and Giuseppe Cesare Gatto worked on database organization and data collection. Eduardo Barbosa Coelho contributed to writing the paper and data analysis. Rafael Freitas de Morais, Leonardo Nunes Alegre and Cesar Perdomo Purper were also involved with database organization. Gabriel Cardozo Muller contributed to database organization and statistical analysis. Rodrigo Citton Padilha dos Reis, Joaquim Francisco dos Santos Neto, Andresa Fontoura Garbini, Veridiana Baldon dos Santos, Rafaela dos Santos Charão de Almeida, Fernanda Costa dos Santos, Raquel Lutkmeier and Daniela dos Reis Carazai contributed with data collection. Rogério Farias Bitencourt, Adriana Melo Teixeira and Olavo Braga Neto contributed to the organization of the group of researchers. Virgínia Angélica Silveira Reis, Flávio Clemente Deulefeu, Fernanda Gadelha Severino, José Gustavo da Costa Neto and Nirvania do Vale Carvalho participated with data collection and database organization and analysis. Finally, André Jamson Rocha de Andrade contributed with data collection and analysis.

Funding

The WHO Global Clinical Platform for COVID-19 is supported by WHO (WHO Voluntary Contributions - Emergencies - Grant: 71856-01 Clinical Care).

Conflict of interests

The authors declare that they have no conflict of interest with the subject of the referred protocol.

References

1. Wu F, Zhao S, Yu B, Chen YM, Wang W, Song ZG, et al. A new coronavirus associated with human respiratory disease in China. Nature. 2020;579(7798):265–9.
2. World Health Organization. Coronavirus Disease 2019. Situat Rep [Internet]. 2020;54:1–9. Available from: https://www.who.int/docs/default-source/coronaviruse/situation-reports/20200314-sitrep-54-covid-19.pdf
3. Roussel Y, Giraud-gatineau A, Jimeno M, Rolain J. SARS-CoV-2: fear versus data. nternational J Antimicrob Agents. 2020;55(January):1–3.
4. Shen Y, Cui Y, Li N, Tian C, Chen M, Zhang YW, et al. Emergency responses to Covid-19 Outbreak: experiences and lessons from a general hospital in Nanjing, China. Cardiovasc Intervent Radiol [Internet]. 2020;43(6):810–9. Available from: https://doi.org/10.1007/s00270-020-02474-w
5. Li T. Diagnosis and clinical management of severe acute respiratory syndrome Coronavirus 2 (SARS-CoV-2) infection: an operational recommendation of Peking Union Medical College Hospital (V2.0): Working Group of 2019 Novel Coronavirus, Peking Union Medical Colle. Emerg Microbes Infect. 2020;9(1):582–5.
6. Cimerman S, Chebabo A, Cunha CA da, Rodríguez-Morales AJ. Deep impact of COVID-19 in the healthcare of Latin America: the case of Brazil. Brazilian J Infect Dis. 2020;24(2):93–5.
7. Barberia LG, Gómez EJ. Political and institutional perils of Brazil's COVID-19 crisis. Lancet. 2020;396(10248):367–8.
8. Gonçalves MR, Padilha dos Reis RC, Tólio RP, Pellanda LC, Schmidt MI, Katz N, et al. Social distancing, mask use, and transmission of severe acute respiratory syndrome Coronavirus 2, Brazil, April–June 2020. Emerg Infect Dis. 2021;27(8):2135–43.
9. Flaxman S, Mishra S, Gandy A, Unwin HJT, Mellan TA, Coupland H, et al. Estimating the effects of non-pharmaceutical interventions on COVID-19 in Europe. Nature. 2020;584(7820):257–61.

For peer review only - http://bmjopen.bmj.com/site/about/guidelines.xhtml

10. Walker PGT, Whittaker C, Watson OJ, Baguelin M, Winskill P, Hamlet A, et al. The impact of COVID-19 and strategies for mitigation and suppression in low- and middle-income countries. Science (80-). 2020;369(6502):413–22.

11. Federação Brasileira de Hospitais, Confederação Nacional de Saúde. Cenário dos hospitais no Brasil 2018 [Internet]. 2018. 108 p. Available from: http://fbh.com.br/wp-content/uploads/2020/11/1-Relatorio-FBH-CNS_web.pdf

12. Ministério da Saúde B. Cadastro Nacional de Estabelecimentos de Saúde [Internet]. 2021. Available from: http://cnes.datasus.gov.br/Cabecalho_Reduzido_Competencia.asp?VCod_Unidade=3205304044916

13. Cotrim Junior DF, Cabral LM da S, Machado FR de S. Fila única de leitos: urgência, dificuldades e enfrentamentos. P2P E Inovação. 2020;7:212–29.

14. Ranzani OT, Bastos L, Gelli J, Marchesi J, Baião F, Hamacher S, et al. Characterisation of the first 250,000 hospital admissions for COVID-19 in Brazil: a retrospective analysis of nationwide data. Lancet Respir Med 2021; 2020;9(January):407–18.

15. Claro IM, Cristina F, Ramundo MS, Candido DS, Silva CAM, Jesus JG De, et al. Local transmission of SARS-CoV-2 Lineage B.1.1.7, Brazil, December 2020. Emerg Infect Dis [Internet]. 2021;27(3):2020–2. Available from: www.cdc.gov/eid

16. da Silva MS, Demoliner M, Hansen AW, Gularte JS, Silveira F, Heldt FH, et al. Early detection of SARS-CoV-2 p.1 variant in Southern Brazil and reinfection of the same patient by p.2. Rev Inst Med Trop Sao Paulo. 2021;63:1–8.

17. Franceschi VB, Caldana GD, de Menezes Mayer A, Cybis GB, Neves CAM, Ferrareze PAG, et al. Genomic epidemiology of SARS-CoV-2 in Esteio, Rio Grande do Sul, Brazil. BMC Genomics. 2021;22(1):1–17.

18. Zeiser FA, Donida B, da Costa CA, Ramos G de O, Scherer JN, Barcellos NT, et al. First and second COVID-19 waves in Brazil: A cross-sectional study of patients' characteristics related

For peer review only

to hospitalization and in-hospital mortality. Lancet Reg Heal - Am. 2022;6(November 2021):100107.

19. Brasil M da S do. DATASUS - Ministério da Saúde [Internet]. Datasus. 2021 [cited 2021 Aug 30]. Available from: www.datasus.gov.br

20. Brasil P da R. Lei Geral de Proteção de Dados Pessoais (LGPD). Diário oficial da união [Internet]. 2018; Available from: file:///C:/Users/usuario/Desktop/L13709compilado.html

21. Merianos A, Peiris M. International Health Regulations (2005). Lancet. 2005;366(January):1249–51.

22. Bartsch SM, Ferguson MC, McKinnell JA, O'shea KJ, Wedlock PT, Siegmund SS, et al. The potential health care costs and resource use associated with COVID-19 in the United States. JAMA J Am Med Assoc. 2020;39(6):927–35.

23. Glied S, Levy H. The potential effects of coronavirus on national health expenditures. JAMA - J Am Med Assoc. 2020;323(20):2001–2.

24. Twohig KA, Nyberg T, Zaidi A, Thelwall S, Sinnathamby MA, Aliabadi S, et al. Hospital admission and emergency care attendance risk for SARS-CoV-2 delta (B.1.617.2) compared with alpha (B.1.1.7) variants of concern: a cohort study. Lancet Infect Dis. 2021;3099(21):1–9.

25. Fiocruz. Observatório Covid-19 [Internet]. https://portal.fiocruz.br/. 2021 [cited 2021 Aug 30]. p. 5. Available from: https://portal.fiocruz.br/sites/portal.fiocruz.br/files/documentos/boletim_covid_2021_extraordinario_30dejunho.pdf

26. Fiocruz. Leitos Uti Covid-19 Para Adultos [Internet]. https://portal.fiocruz.br/. 2021. p. 1. Available from: https://portal.fiocruz.br/sites/portal.fiocruz.br/files/documentos/serie_historica_leitos_uti_covid-19_adultos.pdf

27. Kim MK, Rabinowitz LG, Satish Nagula M, Dunn A, Chalil J, Xu T, et al. A primer for clinician

For peer review only

For peer review only - http://bmjopen.bmj.com/site/about/guidelines.xhtml

deployment to the medicine floors from an epicenter of Covid-19. NEJM Catal. 2020;

28. Berwick DM. Choices for the “new Normal.” JAMA - J Am Med Assoc. 2020;323(21):2125–6.

29. Ortiz-prado E, Simbaña-rivera K, Barreno LG-, Rubio-neira M, Cevallos-robalino D, Sanches-sanmiguel H, et al. Clinical, molecular, and epidemiological characterization of the SARS-CoV-2 virus and the Coronavirus Disease 2019 (COVID-19), a comprehensive literature review. Diagn Microbiol Infect Dis. 2020;98(115094):1–31.

For peer review only

Table 2 - WHO Global Clinical Platform for COVID-19 characterization in Brazil: collaborating centers

STATE	Hospital Institution	Hospital beds							
		Surgical	Clinical	Complementary	Obstetrical	Pediatric	Medical Specialties	Hospital Day	Total
AM	HU Getúlio Vargas (HUGV-UFAM)	58	59	32	0	0	0	0	149
PA	Hospital Universitário João de Barros Barreto (HUJBB-UFPA)	40	178	38	0	28	12	10	306
CE	HU Walter Cantídio (HUWC-UFC)	68	72	21	0	20	22	4	207
CE	Maternidade Escola Assis Chateaubriand (MEAC-UFC)	21	58	65	104	6	0	0	254
CE	Hospital Regional Norte (HRN-ISGH)	58	183	177	33	70	16	0	537
CE	Hospital Geral Waldemar Alcântara (HGWA-ISGH)	18	188	58	0	33	33	0	330
CE	Hospital Regional do Cariri (HRC-ISGH)	88	112	113	0	0	29	0	342
CE	Hospital Regional do Sertão Central (HRSC-ISGH)	58	104	106	30	1	14	0	313
CE	Hospital Estadual Leonardo Da Vinci (Helv-ISGH)	1	112	179	0	0	0	0	292
CE	Hospital Regional Vale do Jaguaribe (HRVJ-ISGH)	0	30	10	0	0	0	0	40
DF	HU da Universidade de Brasília (HUB-UnB)	49	76	41	30	20	3	12	231
ES	HU Cassiano Antônio Moraes (HUCAM-UFES)	82	90	55	20	24	0	2	273
MA	HU da Universidade Federal do Maranhão	134	75	81	71	92	0	0	453
MT	HU Julio Muller (HUJM-UFMT)	12	20	31	24	11	0	0	98
MS	HU Maria Aparecida Pedrossian (Humap-UFMS)	60	45	53	32	18	0	12	220
MS	HU da Univ. Federal da Grande Dourados (HU-UFGD)	29	41	59	25	35	1	0	190
MG	HC da Univ. Federal do Triangulo Mineiro (HC-UFTM)	93	86	57	22	42	2	8	310
MG	HU da Univ. Federal de Juiz de Fora (HU-UFJF)	19	61	29	0	17	0	17	143
MG	HC da Univ. Federal de Minas Gerais (HC-UFMG)	119	114	107	35	63	1	30	469
PB	HU Lauro Wanderley (HULW-UFPB)	31	80	43	36	22	0	33	245
PE	HU da Univ. Fed. Vale do S. Francisco (HU-UNIVASF)	86	35	21	0	0	8	0	150
PE	HC da Univ. Federal de Pernambuco (HC-UFPE)	118	119	27	30	38	13	76	421
RJ	HU Antônio Pedro (HUAP-UFF)	83	54	59	21	17	0	10	244
RN	HU Ana Bezerra (HUAB-UFRN)	8	6	10	33	10	0	0	67
RS	HE da Univers. Feder. de Pelotas (HE-UFPel)	8	109	31	21	20	0	10	199
RS	HU da Univ. Federal de Santa Maria (HUSM-UFSM)	75	163	60	35	41	1	10	385
SP	HU da Univ. Federal de São Carlos (HU-UFSCAR)	0	66	12	0	17	0	0	85

For peer review only

**PB**	**HU Alcides Carneiro (HUAC-UFCG)**	29	84	31	0	33	0	0	177
**PB**	**HU Julio Bandeira (HUJB-UFCG)**	12	2	1	0	15	0	2	32
**RJ**	**HU Gaffree e Guinle (HUGG-UNIRIO)**	95	89	38	19	6	2	3	252
**SE**	**HU da Universidade Federal de Sergipe (HU-UFS)**	49	48	25	0	14	10	10	156
**SE**	**Hospital Universitário de Lagarto (HUL-UFS)**	25	46	33	0	16	0	10	130
**TO**	**Hospital de Doenças Tropicais (HDT-UFT)**	0	37	2	0	9	0	6	54
**BA**	**Hospital Especializado Octávio Mangabeira**	23	105	28	0	17	42	1	216
**PR**	**Complexo Hospitalar do Trabalhador**	126	37	132	35	30	24	0	384
**PR**	**Hospital do Trabalhador**	4	22	0	2	4	0	0	32
**PR**	**Hospital Osvaldo Cruz**	0	29	0	0	1	4	8	42
**DF**	**Hospital da Criança de Brasília José Alencar**	0	0	58	0	150	2	8	218
**PI**	**Hospital Getúlio Vargas**	170	87	94	0	0	2	11	364
**RS**	**Hospital Nossa Senhora da Conceição - Grupo Hospitalar Conceição**	207	357	332	62	105	30	13	1106
**RS**	**Hospital de Clínicas de Porto Alegre - HCPA/UFRGS**	228	224	259	50	95	30	17	903
**SP**	**Instituto de Infectologia Emilio Ribas, São Paulo**	4	82	232	0	16	10	31	375
**SP**	**Hospital Nestor Goulart Reis Americo Brasiliense**	0	20	0	0	0	73	0	93
**Total**		2388	3595	2840	770	1156	384	354	11487

STATE; AM: Amazonas; PA: Pará; CE: Ceará; DF: Distrito Federal; PB: Paraíba; PI: Piauí; RS: Rio Grande do Sul; RN: Rio Grande do Norte; SP: São Paulo; RJ: Rio de Janeiro; BA: Bahia; TO: Tocantins; SE: Sergipe; PA: Pará; PE: Pernambuco; ES: Espírito Santo; MA: Maranhão; MT: Mato Grosso; MS: Mato Grosso do Sul; MG: Minas Gerais.

HU: University Hospital; HE: Teaching Hospital

Source: The Brazilian National Register of Health Service Providers (CNES) (13).

For peer review only

Data Statment Section

Data from all surveyed centers can be aggregated and analyzed – together or by country center or region – to provide a better understanding of the disease and present the public health response to the pandemic via the platform URL: https://www.who.int/teams/health-care-clinic-readiness-unit/COVID-19/dataplatform

For peer review only

Figure 1. Hospitals participating in the survey by size classification

For peer review only

Figure 2. Illustration of flow diagram of study protocol

For peer review only

For peer review only

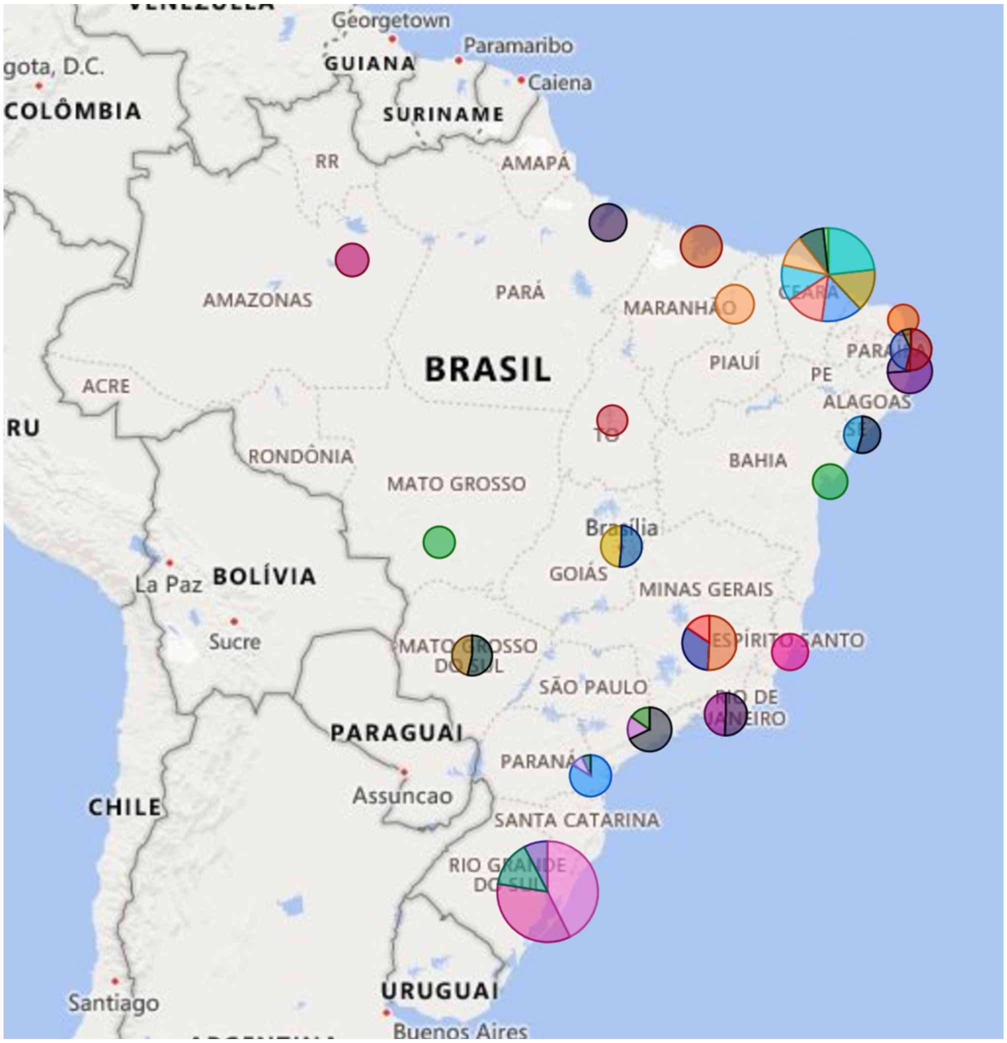

The area of the circle represents the number of beds and the fractions represent the existence of more participating hospitals per state. Source: DATASUS and the Brazilian National Register of Health Service Providers (CNES) (10,21)

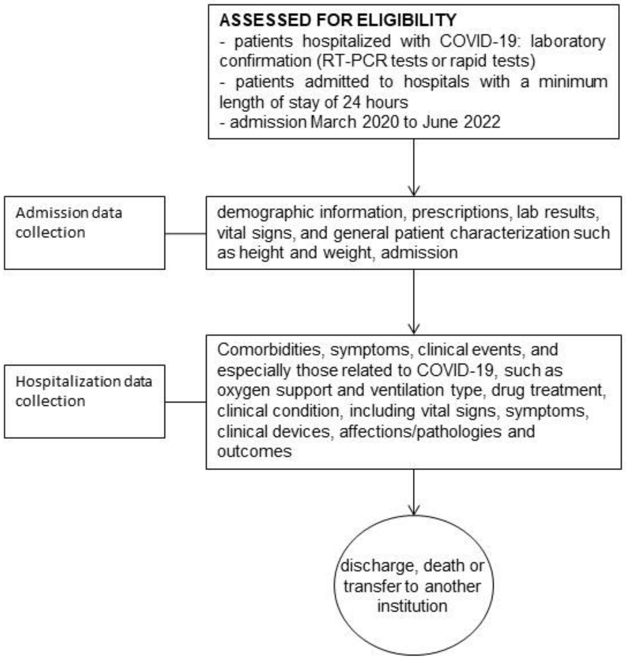

Figure 2. Illustration of flow diagram of study protocol

190x275mm (96 x 96 DPI)

Supplementary 1

Description of the analysis objectives

Objective 1. Description of socio-demographic and clinical characteristics

Socio-demographic and clinical characteristics will be described for the population and pre-specified subgroups. Table 3 presents the description of the study variables with their respective descriptive statistics. Tables and graphs will be used to present the results.

For peer review only

Table 1. Study variables and their values or categories and numerical summary

**Variable**	**Numerical summary**
Age (continuous and categorical)<br>< 18 years old<br>0-4 years<br>5-13 years<br>14-17 years<br>18-45 years<br>46-65 years<br>66-75 years<br>> 75 years old	mean (SD) or median (IQR), when continuous and n/N (%), when categorical
Gender<br>Male, female<br>If female, pregnancy status	n/N (%)
Anthropometry and vital signs on	
admission Temperature (continuous and categorical > 37.5ºC)	mean (SD) or median (IQR), when continuous and n/N (%), when categorical
Heart rate (continuous and categorical > 100 p/ min in adults and adolescents; > 120 in children up to 12 years)	mean (SD) or median (IQR), when continuous and n/N (%), when categorical
Respiration rate (continuous and categorical > 20 r/min in adults and adolescents; > 40 in children up to 12 years)	mean (SD) or median (IQR), when continuous and n/N (%), when categorical
Blood pressure (continuous and categorical systolic > 140 mmHg in adults)	mean (SD) or median (IQR), when continuous and n/N (%), when categorical
Oxygen saturation (continuous and categorical: <90%, <94%)	mean (SD) or median (IQR), when continuous and n/N (%), when categorical

For peer review only

Oxygen saturation in room air/oxygen therapy (categorical)	
Glasgow Coma Scale	mean (SD) or median (IQR)
Height (cm)	mean (SD) or median (IQR)
Weight (kg)	mean (SD) or median (IQR)
BMI (continuous: kg/m² and categorical: ≥ 30; ≥ 40)	mean (SD) or median (IQR), when continuous and n/N (%), when categorical
Obesity (BMI ≥ 30)	n/N (%)
<u>Chronic conditions</u> Chronic heart disease (not hypertension); Diabetes; Hypertension; Current smoker; Chronic lung disease; Tuberculosis; Asthma; Asplenia; Chronic kidney disease; Malignant neoplasm; Chronic liver disease (categorical Yes/No); Other (open text field)	n/N (%)
<u>Pre-admission or chronic medications</u> ACE inhibitors; ARBs; NSAIDs; Antiviral: (if yes, which: chloroquine/hydroxychloroquine, azithromycin, kaletra [lopinavir-ritonavir], favipiravir, Other)	n/N (%)
<u>Signs and symptoms on admission and during hospitalization</u>	n/N (%)
<u>Laboratory examinations on admission and during hospitalization</u> Hemoglobin (g/L); Creatinine (mmol/L); White blood cell count (x10g/L); Sodium (mEq/L); Hematocrit (%); Potassium (mEq/L); Platelets (x10g/L); Procalcitonin (ng/mL); APTT/PT; CRP (mg/L); PT (seconds); LDH (U/L); INR; Creatine kinase (U/L); ALT/SGPT (U/L);	mean (SD) or median (IQR)

For peer review only

Troponin (ng/mL)(mEq/L); Total bilirubin (µmol/L); ESR (mm/hr); AST/SGOT (U/L); D-dimer (mg/L); Urea (mmol/L); Ferritin (ng/mL); Lactate (mmol/L)	
Treatments on admission and during hospitalization	n/N (%)
Antiviral (If Yes, which: Ribavirin, Lopinavir/Ritonavir, Neuraminidase Inhibitor, Interferon alpha, Interferon beta, Other; If Other, specify); Corticosteroids (If Yes, route: Oral, Intravenous, Inhaled; If Yes, specify agent; If Yes, provide maximum daily dose); Antibiotic; Antifungal agent; Antimalarial agent (If Yes, specify); Experimental agent (If Yes, specify); NSAID; ACE inhibitors; ARBs; Systemic anticoagulation	
Examination of the pathogen performed during this disease episode	n/N (%)
Influenza virus (If Positive, specify type); Coronavirus (If Positive, specify type; If Other, specify); Other respiratory pathogen (If Positive, specify type); HIV	
Hospitalization in ICU or High Dependency Unit	
Respiratory and intensive care interventions	n/N (%)
Oxygen therapy; Invasive ventilation (any); Extracorporeal support (ECMO); Liver dysfunction; Pronation; Renal replacement therapy (RRT) or dialysis; Inotropics/vasopressors	n/N (%)<br>n/N (%)

For peer review only

Hospital outcome	
Discharge alive, Hospitalized, Transferred to another unit, Death, Palliative discharge, Unknown	n/N (%)
Complications during hospitalization	
Shock; Bacteremia; Convulsion; Bleeding; Meningitis/encephalitis; Endocarditis; Anemia; Myocarditis/pericarditis; Cardiac arrhythmia; Acute kidney injury; Cardiac arrest; Pancreatitis; Pneumonia; Liver dysfunction; Bronchiolitis; Cardiomyopathy (If Yes, specify)	n/N (%)
Time-to-event outcomes Symptom onset (day of disease on admission): days between first symptom and admission Length of stay Length of stay in ICU Days from hospital admission to ICU admission Days from hospital admission to transfer or death (reported as total, for survivors and non-survivors)	median (IQR), mean (SD), survival curve (Kaplan-Meier)

For peer review only

Objective 2. Characterization of access profiles and hospital resource utilization patterns

The characteristics signs/symptoms, laboratory tests on admission, presence of chronic diseases, treatments received, and outcomes will be described for the population and pre-specified subgroups.

Objective 3. Association between clinical/socio-demographic characteristics and outcomes

Measures of association, such as *odds ratio* (OR), risk ratio (RR) or *hazard ratio* (HR), crude and adjusted for potential confounding variables will be estimated and 95% confidence intervals (95% CI) will be reported. Logistic regression (or Poisson models with robust variance) will be fitted for dichotomous outcomes. Cox proportional hazards models will be fitted for time-to-event outcomes. The assumptions of the regression models will be evaluated using goodness of fit statistics (likelihood and variance analysis) and residual analysis. The set of adjustment variables will be specified primarily by building theoretical models using causal diagrams (*directed acyclic graphs* - DAGs) [5,6].

Objective 4. Sources of inequities

The distribution of socio-demographic, clinical, and outcome characteristics will be described and compared between pre-specified subgroups. Crude association measures (OR, RR or HR, depending on the nature of the outcome) adjusted for potential confounding variables will be estimated and 95% CIs will be reported. The set of adjustment variables will be primarily specified by building theoretical models (DAGs).

For peer review only

STROBE Statement - **Brazilian network for collaborative research on COVID-19: strengthening WHO global data**

	Item No	Recommendation	Page No
**Title and abstract**	1	(*a*) Indicate the study's design with a commonly used term in the title or the abstract<br>(*b*) Provide in the abstract an informative and balanced summary of what was done and what was found	1, 3
**Introduction**			
Background/rationale	2	Explain the scientific background and rationale for the investigation being reported	5-8
Objectives	3	State specific objectives, including any prespecified hypotheses	9, 12
**Methods**			
Study design	4	Present key elements of study design early in the paper	8-17
Setting	5	Describe the setting, locations, and relevant dates, including periods of recruitment, exposure, follow-up, and data collection	10, 12
Participants	6	(*a*) Give the eligibility criteria, and the sources and methods of selection of participants. Describe methods of follow-up<br>(*b*) For matched studies, give matching criteria and number of exposed and unexposed	10
Variables	7	Clearly define all outcomes, exposures, predictors, potential confounders, and effect modifiers. Give diagnostic criteria, if applicable	10,11, 12 and supplementary data
Data sources/ measurement	8*	For each variable of interest, give sources of data and details of methods of assessment (measurement). Describe comparability of assessment methods if there is more than one group	15 and supplementary data
Bias	9	Describe any efforts to address potential sources of bias	11,14
Study size	10	Explain how the study size was arrived at	10
Quantitative variables	11	Explain how quantitative variables were handled in the analyses. If applicable, describe which groupings were chosen and why	supplementary data
Statistical methods	12	(*a*) Describe all statistical methods, including those used to control for confounding<br>(*b*) Describe any methods used to examine subgroups and interactions<br>(*c*) Explain how missing data were addressed<br>(*d*) If applicable, explain how loss to follow-up was addressed<br>(*e*) Describe any sensitivity analyses	11
**Results**			
Participants	13*	(a) Report numbers of individuals at each stage of study—eg numbers potentially eligible, examined for eligibility, confirmed eligible, included in the study, completing follow-up, and analysed<br>(b) Give reasons for non-participation at each stage<br>(c) Consider use of a flow diagram	17
Descriptive data	14*	(a) Give characteristics of study participants (eg demographic, clinical, social) and information on exposures and potential confounders<br>(b) Indicate number of participants with missing data for each variable of interest<br>(c) Summarise follow-up time (eg, average and total amount)	17
Outcome data	15*	Report numbers of outcome events or summary measures over time	17

For peer review only

Main results	16	(*a*) Give unadjusted estimates and, if applicable, confounder-adjusted estimates and their precision (eg, 95% confidence interval). Make clear which confounders were adjusted for and why they were included<br>(*b*) Report category boundaries when continuous variables were categorized<br>(*c*) If relevant, consider translating estimates of relative risk into absolute risk for a meaningful time period	
Other analyses	17	Report other analyses done—eg analyses of subgroups and interactions, and sensitivity analyses	15,16
**Discussion**			
Key results	18	Summarise key results with reference to study objectives	21
Limitations	19	Discuss limitations of the study, taking into account sources of potential bias or imprecision. Discuss both direction and magnitude of any potential bias	18
Interpretation	20	Give a cautious overall interpretation of results considering objectives, limitations, multiplicity of analyses, results from similar studies, and other relevant evidence	19,20
Generalisability	21	Discuss the generalisability (external validity) of the study results	19,20
**Other information**			
Funding	22	Give the source of funding and the role of the funders for the present study and, if applicable, for the original study on which the present article is based	17

*Give information separately for exposed and unexposed groups.

Note: An Explanation and Elaboration article discusses each checklist item and gives methodological background and published examples of transparent reporting. The STROBE checklist is best used in conjunction with this article (freely available on the Web sites of PLoS Medicine at http://www.plosmedicine.org/, Annals of Internal Medicine at http://www.annals.org/, and Epidemiology at http://www.epidem.com/). Information on the STROBE Initiative is available at http://www.strobe-statement.org.

For peer review only
